# Implementation strategies for digital relapse prevention in schizophrenia identified during a randomized controlled trial

**DOI:** 10.1038/s41746-026-03435-2

**Published:** 2026-10-30

**Authors:** Selina Hiller, Katharina Prietzel, Mohamed Shehata, Soňa Nevická Sikorová, Eduard Bakstein, Kateřina Urbanová, Graziella Leone, Laura Emde, Denise Jais, Theodor Lorenzen, Samuel Trumm, Marco Zierhut, Wolfgang Strube, Stefan Leucht, Kerem Böge

**Affiliations:** 1 Technical University of Munich, School of Medicine and Health, Department of Psychiatry and Psychotherapy, TUM University Hospital, Munich, Germany; 2 DZPG (German Center for Mental Health), Partner Site Munich/Augsburg, Munich, Germany; 3 Mindpax.me, Prague, Czech Republic; 4 National Institute of Mental Health, Klecany, Czech Republic; 5 Department of Cybernetics, Faculty of Electrical Engineering, Czech Technical University in Prague, Prague, Czech Republic; 6 Department of Psychology, Faculty of Arts, Charles University, Prague, Czech Republic; 7 kbo-Isar-Amper-Klinikum Region München, München-Haar, Germany; 8 Charité – Universitätsmedizin Berlin, Department of Psychiatry and Neuroscience, Campus Benjamin Franklin, Berlin, Germany; 9 DZPG (German Center for Mental Health), Partner Site Berlin, Berlin, Germany; 10 Berlin Institute of Health at Charité – Universitätsmedizin Berlin, BIH Biomedical Innovation Academy, BIH Charité Clinician Scientist Program, Berlin, Germany; 11 Department of Psychiatry, Psychotherapy and Psychosomatics, Faculty of Medicine, University of Augsburg, Augsburg, Germany; 12 Department for Psychology, Medical University Brandenburg, Neuruppin, Germany; 13 Charité – Universitätsmedizin Berlin, Department of Psychiatry und Psychotherapy, Campus Charité Mitte, Berlin, Germany

**Keywords:** Business and industry, Diseases, Health care, Psychology, Psychology, Scientific community

## Abstract

Digital mental health technologies hold promise, yet their integration into routine care remains challenging. This study reports findings from an implementation workshop conducted midway through an ongoing multi-site randomized controlled trial (RCT) evaluating a smartphone-based relapse prevention app for patients with schizophrenia and schizoaffective disorder. Sixteen stakeholders, including developers, researchers, and clinicians, discussed experiences and barriers using a semi-structured agenda, incorporating prior feedback from three focus groups with people with lived experience, and analyzed the discussions using an adopted R(E)-AIM framework (Reach, Adoption, Implementation, and Maintenance). Findings highlight the need for structural, technical, and personnel adjustments to support sustainable implementation. Key recommendations include integrating digital tools into discharge planning, enhancing interdisciplinary collaboration, delegating digital responsibilities, and streamlining technical workflows to facilitate seamless adoption. These insights inform feasibility considerations and strategic requirements for digital interventions in severe mental illness, offering guidance for future RCTs and practice-oriented integration of digital therapeutics in psychiatric care.

## Introduction

Digital technologies transform mental health care by enabling personalized, scalable, and data-driven approaches to treatment and prevention. They can support early symptom detection, medication adherence, self-management, shared decision-making, and other recovery-oriented outcomes. However, digital tools for severe mental illness (SMI) often show mixed effects in clinical studies^1–8^, and their successful implementation in real-world mental health systems remains inconsistent^6,9,10^. The challenges to move from efficacy in controlled scientific environments to effectiveness in real-world settings are partly rooted in factors operating at different levels of mental healthcare delivery. In particular, factors at the clinician and system level, as well as the patient level, shape the adoption and sustained use of digital interventions. The digital exclusion of people with SMI has declined^11^, and recent studies show high ownership of smartphones and internet access among them^12–14^. Nevertheless, their actual sustainable use of digital devices for mental-health purposes still remains limited^13,15,16^. Various studies report a good feasibility of digital mental health interventions among patients with SMI^4,5,17^. However, engagement rates are reported inconsistently across studies, and usage frequency tends to show a gradual decline over time, despite initial high levels of acceptance and feasibility^6,18,19^. Health-promoting engagement with digital technologies seems to be moderated by various factors such as digital literacy^10,13,20–22^, trust in data security^10,12,16^, intention to use or expected benefit^21,23^, perceived bond with the digital intervention^19^, but also fear of confrontation or even worsening of symptoms^21^. Studies that take into account the effect of engagement when evaluating a digital intervention´s efficacy are rare^24^. Health professionals play an equally important role when it comes to engagement and, in turn, effective implementation of digital interventions. They are central in raising awareness and promoting the uptake of those interventions. Research thus highlights the importance of conceptualizing an optimal referral strategy for suitable patients from respective clinicians^22,25,26^. More importantly, research shows that self-led digital interventions are often prone to discontinuation or suboptimal use, whereas those that involve human support tend to foster adoption and sustain engagement^10^. Surveys frequently report general positive attitudes among healthcare professionals towards the implementation of digital mental health approaches in their clinical practice^27,28^. In real-world settings, however, they frequently perceive digital mental health tools as disruptive rather than supportive, particularly when interventions are misaligned with established routines or fail to demonstrate clear added clinical or economic value^21,29^. They are often discontinued due to lack of sustained clinician engagement, structural support, or necessary systemic infrastructure^9,10,21,26,30–32^.

A growing body of literature thus emphasizes the importance of structured user involvement throughout all stages of the research process to ensure the relevance, acceptability, and efficacy of innovative interventions^18,25,30,33^. Participatory, human-centered- or co-design approaches have become popular strategies in the iterative development and implementation of digital (mental) health applications^34–37^. These concepts are also applied in populations with SMI^38,39^. Above, implementation research aims to analyze contextual necessities across various stakeholder levels for successful translation of scientific innovations into real-world conditions^30,33,40–43^. The development of relevant digital mental health applications requires successful inter- and transdisciplinary collaboration to create applications that are not only technically sophisticated, but also effective in a clinical sense^33,43,44^. Core frameworks such as the Consolidated Framework for Implementation Research (CFIR)^43,45–47^ or the Reach, Effectiveness, Adoption, Implementation and Maintenance Framework (RE-AIM)^48–51^ offer structured approaches to identify and evaluate determinants of implementation success across multiple levels. The frameworks are also being more widely adopted in digital mental health research^21,43,52,53^. Many implementation studies, however, apply these frameworks to map well-researched implementation determinants of digital interventions retrospectively. Contemporary approaches increasingly advocate a non-linear translational pathway, i.e., embedding implementation considerations throughout all phases of intervention development and evaluation^33^. Hybrid effectiveness–implementation trials are specifically designed to evaluate clinical efficacy, while simultaneously examining implementation processes along these established implementation science frameworks^41^.

This article presents outcomes from a mid-study pragmatic implementation evaluation of a digital mental health intervention, conducted within the context of an ongoing multi-site randomized controlled trial (RCT; ILIA trial; German Clinical Trials Register DRKS00034991). The primary objective of the ILIA trial is to evaluate the effectiveness of a digital relapse prevention intervention on hospitalizations and other relevant clinical outcomes for individuals with schizophrenia or schizoaffective disorder^54^, while we additionally gathered information on implementation barriers and facilitators. With that, this study falls under the formal definition as a Type 1 hybrid effectiveness–implementation trial^55^. The primary data collection instrument of the following article was a structured qualitative stakeholder workshop, in which interdisciplinary participants reviewed and discussed implementation experiences using an adopted R(E)-AIM framework as an organizing scaffold^49,51^. We begin this article by describing the characteristics of the stakeholder workshop participants, followed by a structured presentation of results organized according to four of the five R(E)-AIM dimensions. Results on the Effectiveness will be published upon RCT completion. Within each dimension, we report the implementation strategies enacted during the trial, alongside the facilitators and barriers identified through our qualitative-descriptive analysis of the workshop data. The discussion section then situates these findings within the broader literature on digital mental health implementation in SMI and derives implications for future implementation efforts. The methods section at the end of the article describes the pragmatic qualitative-descriptive study design^56,57^ and our analytical approach based on directed content analysis along the R(E)-AIM categories^58^.

## Results

### Workshop participants

On May 26, 2025, one semi-structured implementation workshop was held with 16 stakeholders, with an equal distribution between on-site and remote participation. Six clinicians, two principal investigators (S.L. & K.B.), three research associates, two student assistants, and three representatives from the digital mental health company participated. The workshop was moderated by a female member of the study team (S.H., MSc Health Sciences, with prior experience in qualitative research), who also performed the qualitative analysis and had prior working relationships with several participants through the trial. This familiarity may have facilitated open discussion but may also have shaped which topics were raised. In addition, three co-authors affiliated with the intervention provider participated in the workshop as one stakeholder group.

The workshop lasted 3 h and 40 min. Identifiers are used consistently across all sections, and participants may appear more than once if they contributed comments relevant to multiple themes. Quotations are illustrative exemplars of consolidated themes. All reported themes were corroborated across the collaboratively generated worksheet data and the workshop transcript and do not represent isolated individual viewpoints. Quotations are labeled with participant pseudonyms (P1–P16) consistent with the coded workshop transcript. The quoted participants were selected from the 16 stakeholders for clarity of articulation and therefore do not appear in consecutive numerical order in the results section.

### Workshop discussions structured along the adopted R(E)-AIM framework

We adopted the original RE-AIM framework and included four of the five domains within our analysis (R(E)-AIM). Within each domain, results are classified as barriers (factors that impeded implementation) or facilitators (factors that promoted implementation) and, if applicable, implementation strategies presented. This approach follows recent guidance on analytical RE-AIM application^49,51,59^, in which the framework not only serves as a classification system, but as an explanatory tool for understanding the determinants of implementation outcomes. In the following, Reach is examined through a reflection on recruitment initiatives and outcomes drawing on study sample data. Adoption is evaluated using a description of pre-trial implementation strategies, followed by an in-depth discussion of six purposively selected patient use cases, chosen to illustrate varying degrees and contextual circumstances of adoption. The dimensions of Implementation and Maintenance are then addressed primarily through thematic findings from open stakeholder workshop discussions. Table 2 includes the main results structured along those dimensions.

### Reach

To facilitate our recruiting strategy, we conducted kick-off meetings for clinical teams at each of the eight participating study centers prior to the recruiting phase (*n* = 8), each lasting approximately 1 h. These site-specific sessions introduced the ILIA study design, including inclusion criteria, recruitment procedures, and the digital intervention. Further, bi-monthly newsletters provided the current recruitment status and other relevant study updates for participating clinicians. The planned sample size was 110 participants based on the sample size calculation^54^. Recruitment continued beyond this target, resulting in a final enrolled sample of 115 participants. At the time of the implementation workshop, 77 participants had been recruited. The majority were existing patients of the participating outpatient clinics, facilitating enrollment and initial engagement. Patients in stable chronic conditions and individuals with significant cognitive impairments, no digital literacy, or lacking reliable internet access were excluded^54^. To further facilitate recruitment, we tried to minimize the digital divide by providing study smartphones to participants who did not own a smartphone, but with at least basic digital skills and regular access to the internet. Of the reported screening failures, having no interest in study participation (*n *= 55), not meeting the inclusion criteria because of a wrong diagnosis (*n *= 52) or being too stable (*n *= 48), were among the most common reasons for not participating in the study. Among patients who met the digital eligibility criteria, skepticism towards digital monitoring was rarely the stated reason for declining participation (skeptical *n *= 7; not using digital devices *n *= 12). Because patients without digital literacy or reliable internet access were excluded by protocol, these figures indicate only that, within the eligible group, digital skepticism was not a major stated barrier to participation. We asked clinicians to report screening failures proactively. We nevertheless followed up occasionally but cannot ensure that the reported numbers represent the complete set of screening failures across these centers.

Descriptive statistics for the study sample are presented in Table 1. The mean age of participants was 39.06 years (SD = 11.35; *n *= 115). Clinician-reported duration since first diagnosis averaged 148.77 months (SD = 133.87; *n *= 109), indicating that, on average, the sample comprises individuals with a rather chronic illness, while the substantial variability suggests that some participants were diagnosed more recently. Patients reported an average of 6.54 previous episodes (SD = 13.15; *n *= 108), while clinicians reported 4.84 episodes (SD = 4.37; *n *= 109). Patients indicated they had spent a mean of 66.75 days in hospital over the past 2 years (SD = 84.22; *n *= 112), and clinician reports showed a comparable estimate of 67.48 days (SD = 85.15; *n *= 86). Clinician reported days in hospital in the last 2 years are missing for 29/115 participants (25.22%), predominantly where clinicians did not have complete records of prior admissions available at baseline. Missingness was concentrated at one site (14/27, 51.85%, vs. 15/88, 17.05%, across the remaining sites), reflecting that this site additionally recruited external patients newly enrolled for the study without prior attachment to its outpatient clinic, for whom clinicians sometimes had no records of prior admissions available at baseline. This variable is reported for descriptive purposes only and does not enter any analysis. The level of recent service use and hospitalizations aligns with the relapse-prevention focus of the intervention. Symptom severity at baseline, assessed with the PANSS-6^60^, showed a mean total score of 14.10 (SD = 5.98; *n *= 113), corresponding to mild to moderate levels of symptomatology at enrollment. Clinical global severity (CGI-S^61^) at baseline was rated on average as 3.96 (SD = 0.91; *n *= 112), consistent with moderate symptom levels. Patient-reported digital health literacy (eHEALS total score^62^) averaged 28.65 (SD = 6.72; *n *= 115), indicating moderate to high levels of perceived literacy. Twenty-six participants (22.61%) reported a foreign nationality, which is broadly consistent with the proportion of individuals with a migration background in the general population, supporting the ecological validity and indicating a certain degree of generalizability of the sample. Forty-seven individuals (40.87%) identified as female. Just over half of the sample (61 participants, 53.04%) reported a high school diploma as their highest level of education (*n *= 115 for all categorical variables).

**Table 1:** Baseline participant data

Variable numeric	Missing (*n*)	Mean (SD)
Age	0	39.06 (11.35)
Duration since first diagnosis in months (clinician-reported)	6	148.77 (133.87)
Number of previous episodes (patient reported)	7	6.54 (13.15)
Number of previous episodes (clinician reported)	6	4.84 (4.37)
Days in hospital in the last 2 years (patient reported)	3	66.75 (84.22)
Days in hospital in the last 2 years (clinician reported)	29	67.48 (85.15)
PANSS-6^60^ at baseline (clinician reported)	2	14.10 (5.98)
CGI-S^61^ at baseline (clinician reported)	3	3.96 (0.91)
Patient digital literacy (eHEALS total score^62^)	0	28.65 (6.72)
**Variable dichotomous**		***n*** **(%)**
Foreign nationality	0	26 (22.61%)
Female	0	47 (40.87%)
High School Diploma as highest education	0	61 (53.04%)

**Fig. 1:**
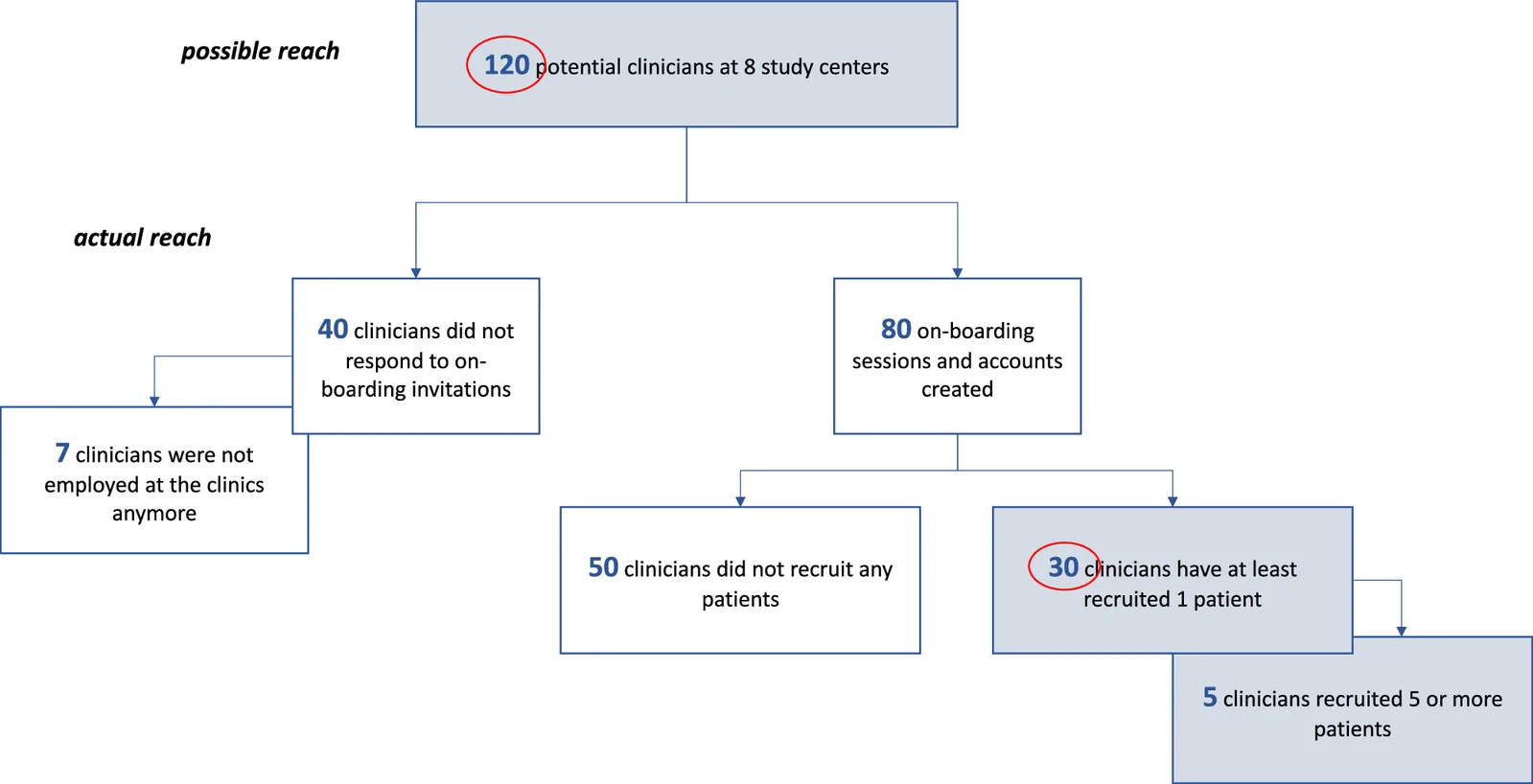
Clinician recruitment flow across eight study centers during the study enrollment phase. Of 120 potential clinicians identified (possible reach, red circle), 40 did not respond to on-boarding invitations. 80 clinicians completed an on-boarding session (actual reach). Of those, 50 did not recruit any patients, whereas 30 recruited at least one patient (red circle). A subset of 5 clinicians (shaded box) recruited five or more patients. Data reflect the end of the recruiting phase in July 2025.

Internal recruitment targets indicated that up to 120 clinicians could potentially have been involved across the eight participating centers. Of the 120 clinicians, two-thirds (*n *= 80) responded and were present at a kick-off or individual on-boarding session (Fig. 1). Of those 80 clinicians, 37.5% ended up recruiting at least one patient at the end of the recruiting period. The other 50 clinicians either did not respond to further recruitment inquiries or only reported screening failures. A major barrier for clinician engagement in the ILIA study was that clinicians were not directly incentivized for their contribution to recruitment and had to implement the intervention alongside their regular duties as resident clinicians.

One workshop participant noted that patient benefits were not sufficiently clear, which may have hindered clinicians’ engagement in recruitment. According to her, perceived extra workload, limited immediate benefit, and concerns about privacy and transparency due to digital data collection were a main barrier to recruitment and likely hindered broader participation:

***P7:*** *“Well, I know some colleagues who had some reservations about these digital measures—the ‘transparent patient,’ now they’re being monitored in some way, and, yeah, I think the benefits weren’t entirely clear.”*

### Adoption

We defined Adoption as the usage patterns of patients and clinicians. For adequate adoption, we implemented two major strategies prior to clinical trial start. During the workshop, we then explored the actual adoption through the discussion of six illustrative use cases across study sites and identified major facilitators and barriers to adoption.

Proper onboarding of both patients and clinicians is a major facilitator to improve adoption of innovative interventions^22,42^. The previously mentioned kick-off meetings additionally served as an early opportunity to gather feedback from clinical teams. During one of the kick-off meetings, it was suggested that alert notifications should not only be sent to the designated clinician, but to all clinicians in the outpatient team, including psychiatrists, psychologists, psychiatric nurses, and others. The rationale was to ensure continuity of care in case of part-time schedules, vacation, or sick leave. Based on this input, the notification logic was technically adapted before study launch. Between September 2024 and February 2025, additional individual 1:1 onboarding sessions were conducted with respective clinicians (*n *= 80), each lasting approximately 30 min. We set up accounts in the digital clinic portal and provided step-by-step guidance for interacting with the digital platform. In total, 80 onboarding sessions with clinicians were conducted across all sites, and their accounts were created and activated. Above, we created a set of concise support materials, as proposed in previous studies on potential preparation measures for a seamless adoption and implementation^25,42^. Clinicians received quick-reference handouts that summarized key onboarding content along with visual process flow diagrams. Patients received a concise reference sheet that included the login procedures and contact details of study coordinators. To relieve clinicians of troubleshooting and procedural responsibilities, the ILIA study team was the primary point of contact for any technical issues or study-related questions.

**Fig. 2:**
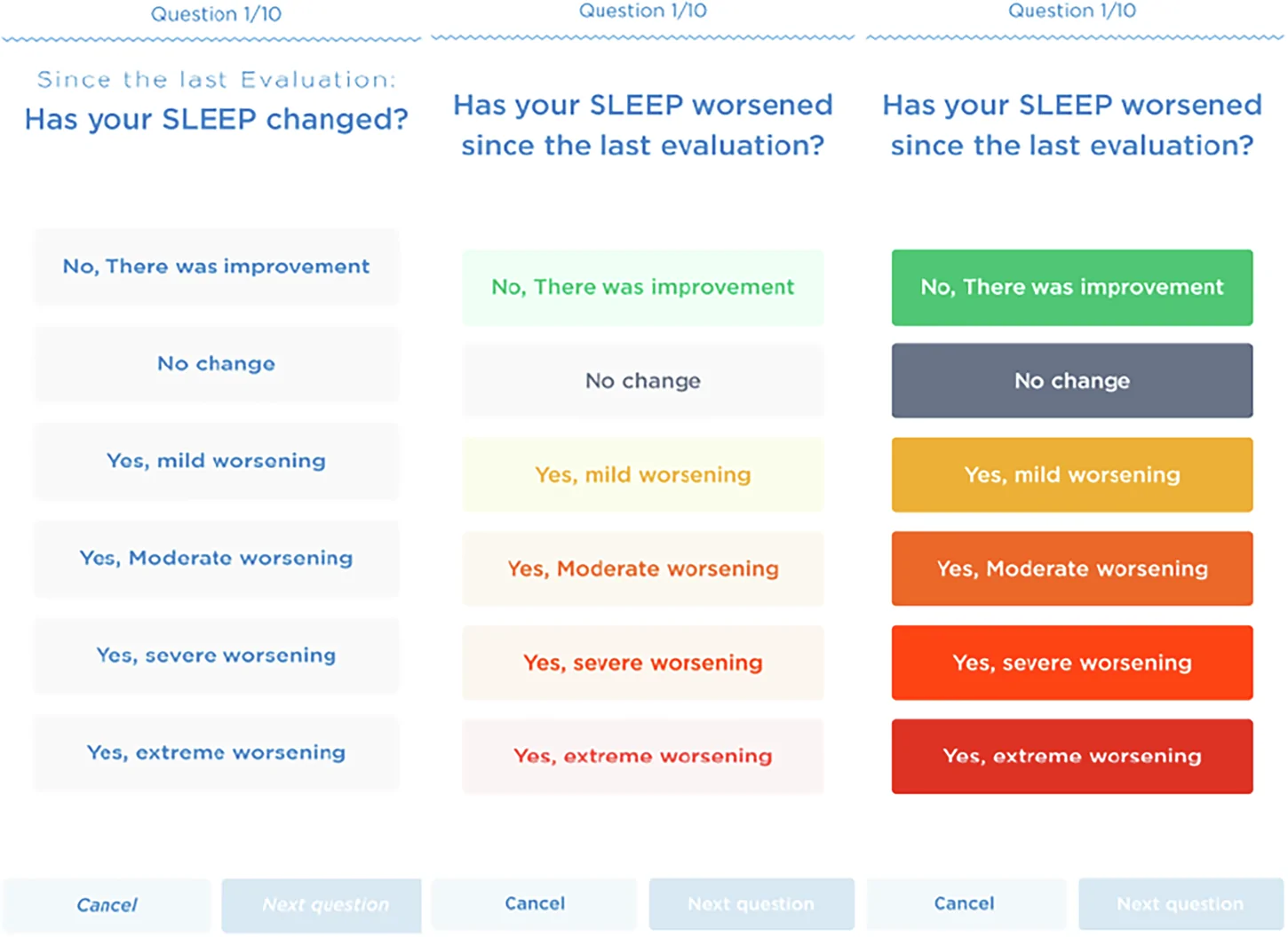
Three color scheme variants for the weekly monitoring questionnaire interface evaluated during participatory focus groups. Three design options were presented to participants during co-design activities. A neutral uncolored variant (left) and two color-coded variants using a green-yellow-red gradient to distinguish response categories ranging from improvement to severe worsening (center, right). Based on participant feedback, the transparent color-coded design was selected and implemented in the final app.

Above, three exploratory hybrid pre-RCT focus groups with patient representatives were conducted on 02 February 2024, 08 March 2024, and 18 April 2024 to support peer-informed refinement of the intervention design. We initially recruited seven persons with lived experiences using a convenience sampling approach via the German national BASTA e.V. network (https://www.bastagegenstigma.de/), an advocacy organization dedicated to combating stigma and discrimination against people with mental illness. The same seven participants were invited to all three focus groups to ensure continuity across iterative development stages, although participation was reduced in the second (*n *= 5) and third (*n *= 3) focus groups. Each session lasted approximately 1 h and was facilitated by at least two researchers, with one acting as moderator and the other responsible for structured protocol note-taking. These notes captured key statements and discussion points verbatim or in summarized form. The documented material was not formally analyzed using a predefined qualitative coding framework. Instead, an exploratory synthesis approach was applied to identify actionable insights for iterative intervention development. Across the focus groups, participants provided valuable feedback on app usability, appropriate language use, and concerns related to digital monitoring. Insights from these sessions informed subsequent modifications to the app design. One valuable participatory adaptation concerned the visual design of the app-based relapse questionnaire. Focus group participants were presented with the original non-colored version alongside two alternative color schemes. In a joint discussion, the group decided on a transparent red–yellow–green gradient to intuitively represent increasing levels of symptom severity. This color scheme was subsequently implemented in the final version of the app (Fig. 2).

**Fig. 3:**
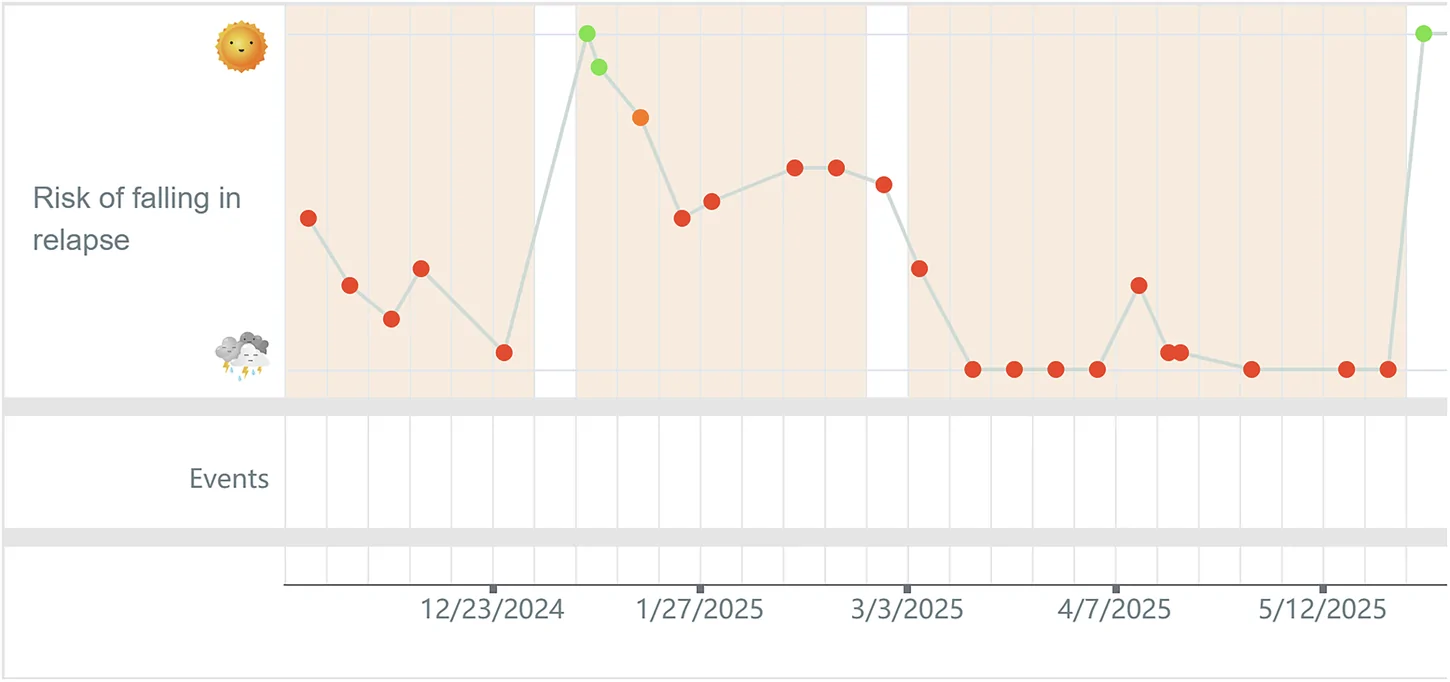
Visualized symptom trajectory of a patient during a persistent alert period in Use Case 1. Calendar weeks are represented by vertical bars, and individual data points correspond to weekly questionnaire responses, projected onto a relapse risk score via an internal algorithm. Orange-shaded rectangles indicate alert periods. Data points reflect weekly EWSQ-10P responses projected onto a relapse risk score ranging from low (sun icon) to high (storm cloud icon).

Use case 1 addressed challenges of self-assessment and app engagement during a persistent alert period. At the time of the workshop, the patient was enrolled for 6.2 months (study entry date 19.11.2024), the system has triggered 13 alert periods or worsening during an ongoing alert period. There were 14 patient contacts (i.e., not reached, reached by phone, or action taken) entered into the web-based portal post-alert by the respective clinician. The patient completed weekly symptom assessments regularly but remained in a continuous alert period triggered by self-reported deteriorations across multiple categories (Fig. 3). Each interaction between the patient and the responsible clinician was documented in the clinician’s digital portal. Check-in calls revealed that the patient wanted to document minor deteriorations and did not feel at risk of relapse. From an implementation perspective, this case illustrates how a mismatch between the intervention’s algorithmic logic and the patient’s subjective illness appraisal can generate clinically misleading outputs and be a barrier to adoption. This use case highlights the importance of adequate onboarding procedures, patient education, and transparency to facilitate the intended interpretation of self-assessment tools.

**Fig. 4:**
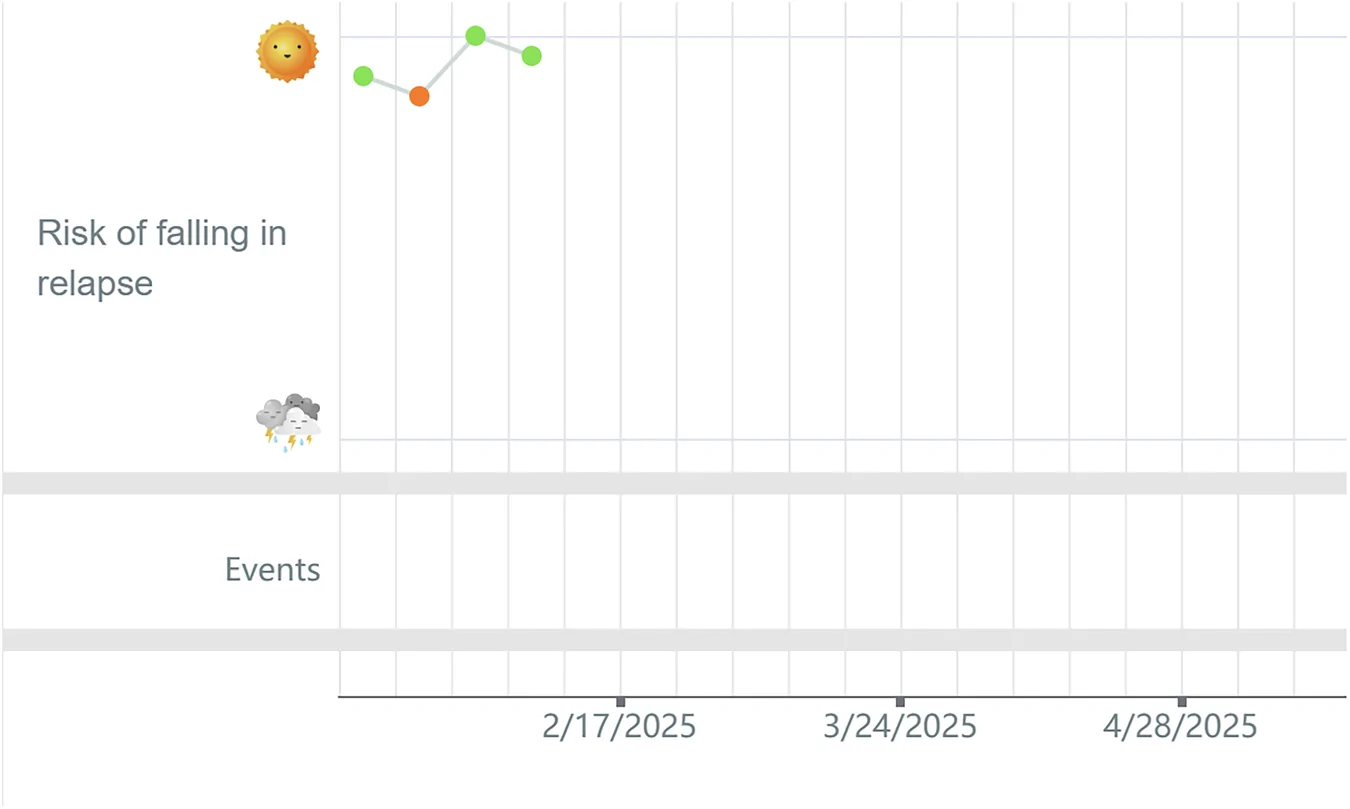
Symptom monitoring trajectory of a representative patient who discontinued app use after four entries in Use Case 2. Individual data points reflect weekly questionnaire responses (EWSQ-10P), projected onto a relapse risk score ranging from low (sun icon) to high (storm cloud icon) via an internal algorithm. The patient completed only four assessments in early 2025 before ceasing all app engagement, with no subsequent medication logs or life events recorded.

Use case 2 focused on an acute relapse following prolonged app inactivity and failed contact attempts. At the time of the workshop, the patient was enrolled for 4.3 months (study entry date 16.01.2025), the system has triggered no alert periods, and 8 patient contacts have been entered into the web-based portal post-alert by the respective clinician. Contact attempts were documented because the patient stopped using the app due to technical issues (broken phone and forgotten password). Despite verbal agreements with study personnel to resume usage, engagement did not recover (Fig. 4). The patient then experienced a relapse requiring hospitalization that went digitally undocumented, revealing the vulnerability of digital monitoring in case of non-adherence. From an implementation perspective, this case highlights the facilitating role of patient-endorsed passive monitoring options and secondary contact strategies to maintain clinical visibility and continuous adoption. Backup communication strategies, such as caregiver contact, can serve as key facilitators for continuity of adoption. The case also highlights prolonged app inactivity as an adoption-level phenomenon and a potential future analytic signal warranting follow-up. Whether such disengagement reliably indicates symptom worsening is an effectiveness question to be examined on completion of the RCT analysis.

**Fig. 5:**
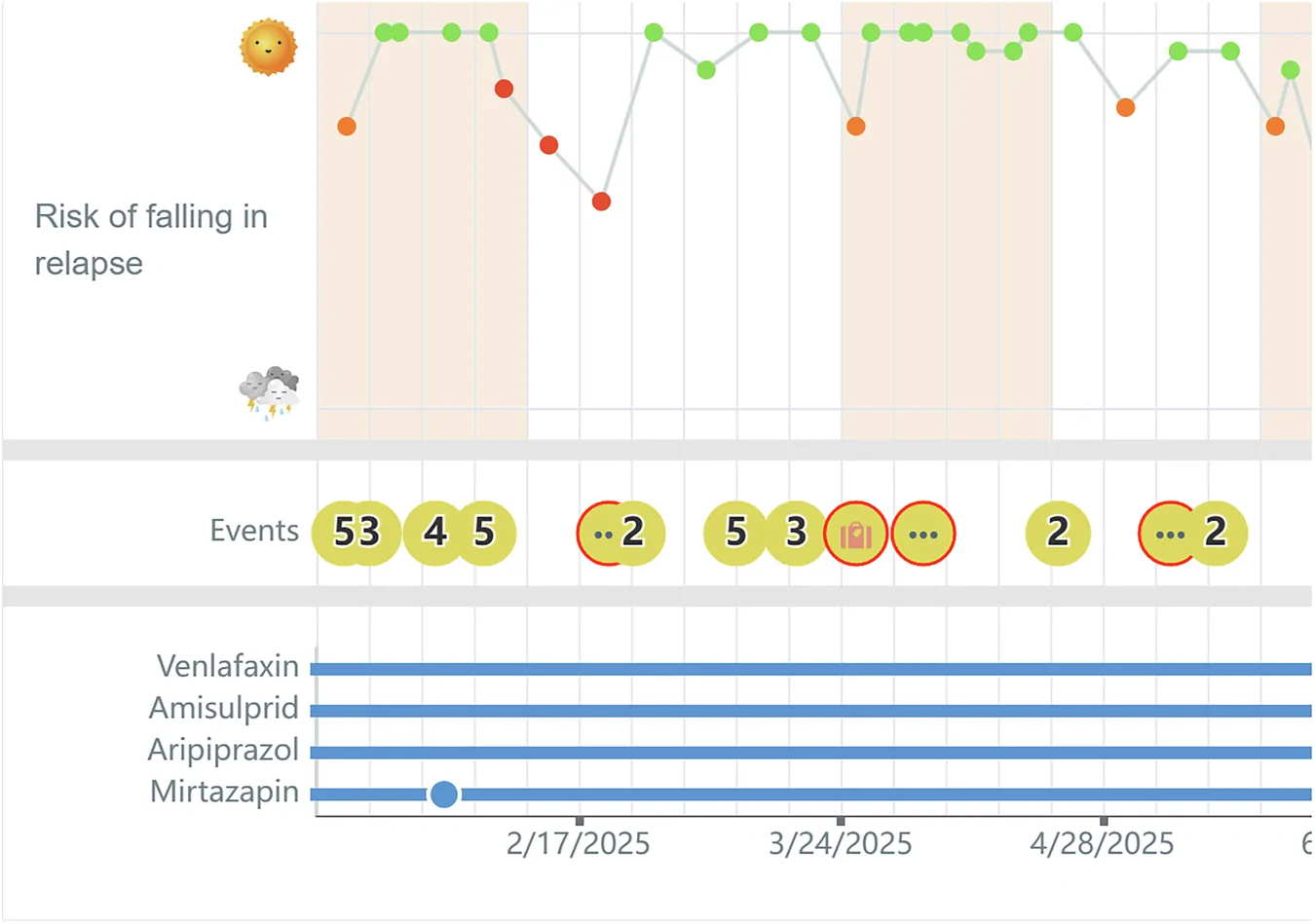
Frequent app engagement with regular life events and medication logging in a highly active patient in Use Case 3. Data points reflect weekly EWSQ-10P responses projected onto a relapse risk score ranging from low (sun icon) to high (storm cloud icon). Orange-shaded rectangles indicate alert periods. The patient demonstrated consistently high app usage throughout the observation period, frequently documenting life events and medication changes, illustrating how reassurance-seeking behavior may drive increased engagement with digital symptom monitoring.

In use case 3, reassurance-seeking behavior was associated with increased app usage. At the time of the workshop, the patient was enrolled for 4.3 months (study entry date 16.01.2025), the system has triggered 5 alert periods or worsening during an ongoing alert period, and 1 patient contact has been entered into the web-based portal post-alert by the respective clinician. Overall, the patient used the app regularly, documented personal events, and contacted the study team frequently (Fig. 5). According to the treating clinician, the patient is highly active in managing her chronic diseases, including frequent reassurance-seeking contact, self-education, peer groups, and maintaining social connections. The app offered the patient a structured channel for self-monitoring and documentation. From an implementation perspective, this case represents a positive adoption trajectory facilitated by intrinsic motivation, illness identity integration, and an existing social support network. It illustrates the facilitating role of patient-level activation as a key facilitator of sustained adoption.

**Fig. 6:**
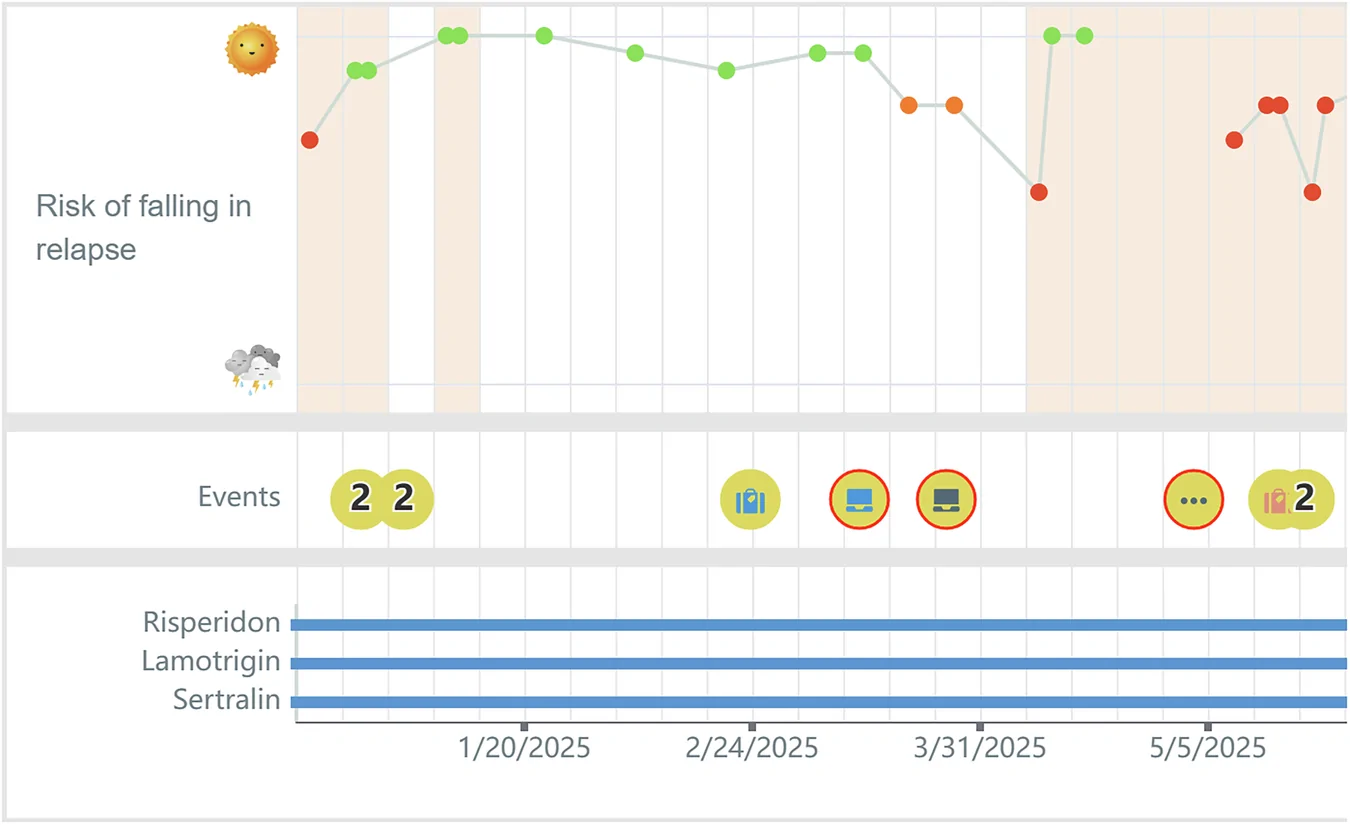
Stable symptom trajectory with good overall app adherence but reduced engagement during symptom-free phases in Use Case 4. Data points reflect weekly EWSQ-10P responses projected onto a relapse risk score ranging from low (sun icon) to high (storm cloud icon). Orange-shaded rectangles indicate alert periods. The patient demonstrated consistent app use, including regular medication and life event logging, throughout the observation period but showed reduced questionnaire completion during clinically stable phases.

In use case 4, disengagement during stable phases was observed. At the time of the workshop, the patient was enrolled for 5.2 months (study entry date 19.12.2024), the system has triggered 4 alert periods or worsening during an ongoing alert period, and 5 patient contacts have been entered into the web-based portal post-alert by the respective clinician. The patient used the app irregularly, particularly during symptom-free phases (Fig. 6). The patient often forgot the app when feeling well or being busy. Despite built-in push notifications and reminder phone calls by the study team, the patient’s well-being and life demands led her to deprioritize the app. From an implementation perspective, this case maps from the adoption onto the maintenance dimension and highlights perceived relevance as a critical and variable implementation determinant. Adoption appeared contingent on the patient’s current symptom burden rather than on a stable understanding of the tool’s preventive function, pointing to a gap in psychoeducation around the early-warning logic of the intervention. Relevant implementation facilitators include the design of engagement-sustaining features, the framing of the intervention’s purpose, and the adequacy of reminder strategies in low-symptom phases.

**Fig. 7:**
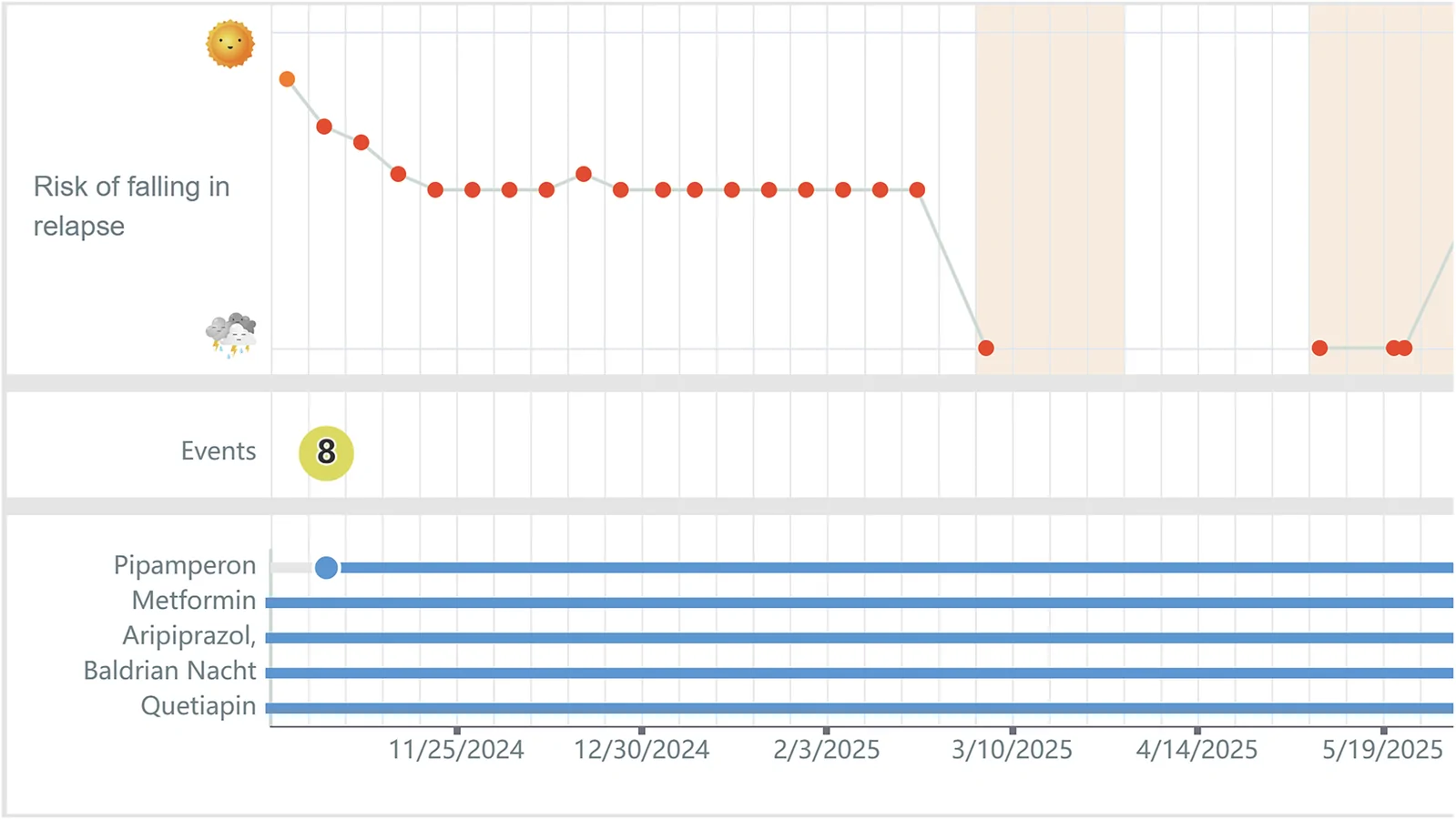
Gradual symptom worsening without alert threshold breach preceding non-response and hospitalization in Use Case 5. Data points reflect weekly EWSQ-10P responses projected onto a relapse risk score ranging from low (sun icon) to high (storm cloud icon). Orange-shaded rectangles indicate alert periods. Despite weeks of continuous deterioration, the alert threshold was not reached until later in the observation period, after which the patient became unreachable and was subsequently hospitalized.

Use case 5 focused on a patient who became unreachable after symptom worsening. At the time of the workshop, the patient was enrolled for 7 months (study entry date 22.10.2024), the system has triggered 6 (extended) alert periods and 3 patient contacts have been entered into the web-based portal post-alert by the respective clinician. At some point, after declining a proposed hospitalization due to symptom worsening, the patient abruptly stopped using the app (Fig. 7). Contact attempts failed until a family member, listed as emergency contact by the patient at study start, was contacted by the study team and reported a hospital admission. As the patient’s worsening symptoms remained just below threshold, no alert was generated. From an implementation perspective, this case exposes a critical barrier at the intersection of the adoption and maintenance dimensions. The symptom-driven disengagement and an insufficiently sensitive alert threshold produced a situation of clinical invisibility despite ongoing risk. The key implementation determinants are a personalized alert system, the formal integration of relatives as active implementation stakeholders, and the availability of escalation protocols that do not rely solely on patient-initiated contact.

**Fig. 8:**
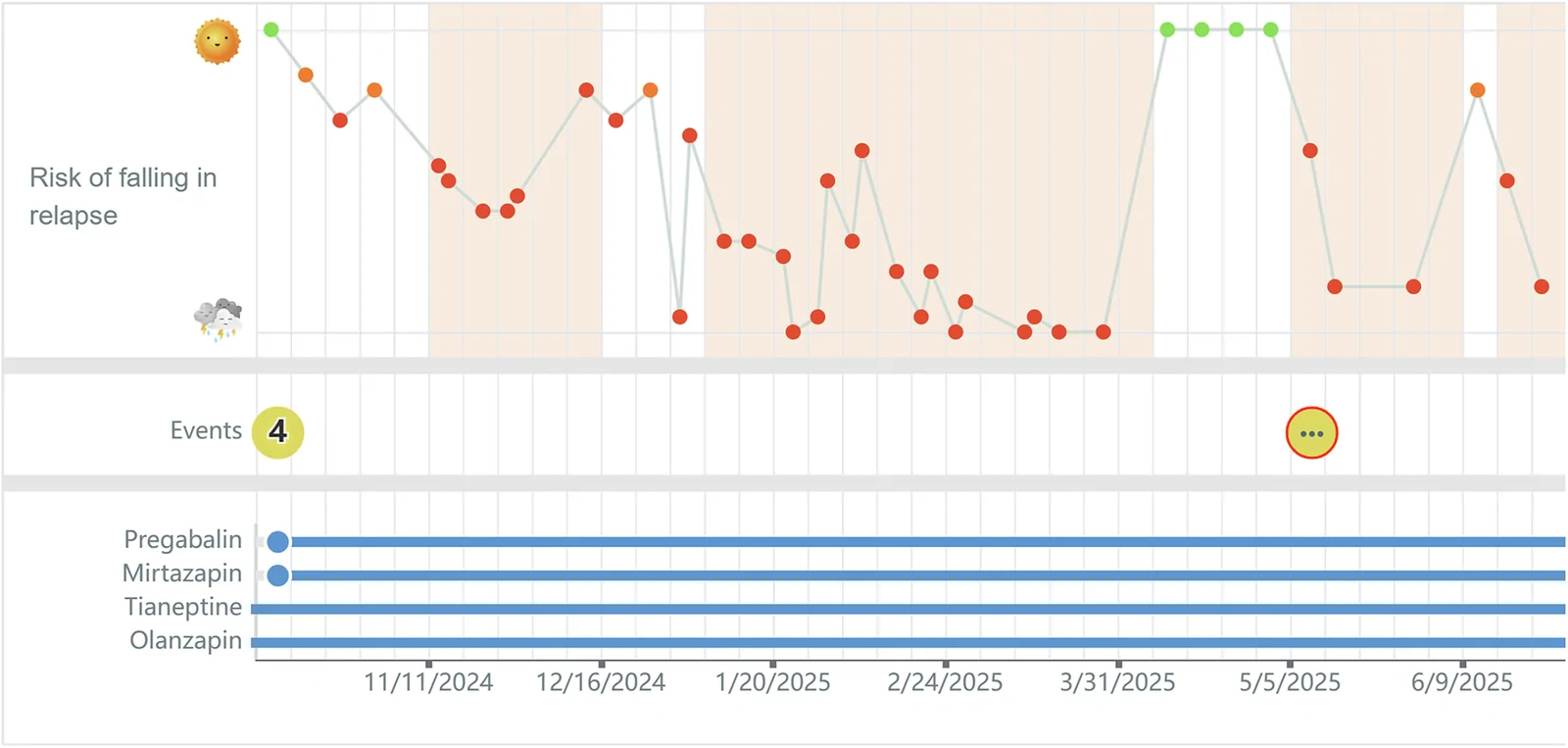
Continuous symptom worsening across multiple alert periods in a representative patient with somatic comorbidities in Use Case 6. Data points reflect weekly EWSQ-10P responses projected onto a relapse risk score ranging from low (sun icon) to high (storm cloud icon). Orange-shaded rectangles indicate alert periods. This case illustrates how somatic comorbidities may distort digital symptom tracking and complicate alert interpretation.

Use case 6 focused on somatic comorbidities that distorted digital symptom tracking. At the time of the workshop, the patient was enrolled for 7.5 months (study entry date 11.10.2024), the system has triggered 18 alert periods or worsening during an ongoing alert period, and 18 reactions have been entered into the web-based portal post-alert by the respective clinician. This patient remained in a constant alert period due to high somatic distress, while psychiatric symptoms were stable (Fig. 8). This interpretation was established through in-person outpatient appointments during the initial study phase, in which the treating clinician was able to verify that the ongoing alerts were attributable to somatic pain, rather than psychiatric deterioration. Subsequently, the patient canceled all study visits, resulting in a situation where digital monitoring continued without any in-person verification. Interestingly, the patient remained digitally present but physically absent from care structures. This case illustrates two distinct but interrelated implementation determinants. Blended care interventions depend on the human care component functioning in parallel; i.e., once the patient withdrew from clinical contact, digital monitoring data became unactionable without the interpretive context that clinician interaction provides. Second, the case highlights the need for algorithm refinement capable of accounting for the diagnostic complexity and somatic comorbidities, common in populations with SMI.

### Implementation and maintenance

During the workshop discussions, two major facilitators for long-term implementation were identified by the participants. Delegating onboarding, training, and app-related tasks to study nurses or digital support staff, as we employed in the ILIA trial, facilitates sustainability without overburdening clinical teams. There was broad agreement among workshop participants that future real-world implementation efforts should involve extended care teams (e.g., nurses, peer support workers, family members) and clear role definitions. For this to work effectively, workshop participants concluded that formal standard operating procedures (SOPs) on the organizational level are needed to facilitate consistent alert management across sites, clinicians and patients. One workshop participant suggested embedding such digital relapse monitoring tools within discharge planning protocols to optimize the reach of suitable patients:

***P14:**** “wouldn’t [it] be best to put the patients on the system prior than leaving the hospital. […] Because you have the staff there, you have the dedicated workshops, right, or the sessions and so on. And you have the time there. And it’s also the most risky period for the next relapse to happen so and also for hospital systems, from the economic perspective, it is much better for the hospital if the patient doesn’t relapse after they disburse him.”*

According to workshop participants, a frequently overlooked facilitator in implementation and maintenance is the involvement of family members and close social contacts. In the ILIA trial, we recorded emergency contacts for participants, if they consented. We contacted them when both clinicians and study staff were unable to reach the patient during an (extended) alert period.

Through discussion of the implementation strategies of the ILIA trial, workshop participants identified numerous barriers for successful long-term implementation. They concluded that limited clinician time and training capacity, alongside insufficient financial incentives and lack of institutional recognition, are the most salient barriers to long-term adoption and sustained clinical engagement. This is highlighted by statements of two of our workshop participants:

***P7:**** “For example, we are a large team, but we are self-funded, so time is definitely a factor—every hour we don’t spend treating patients, of course, we miss out in the end […] Ideally, there should be some dedicated time or a day when we can focus solely on the app.”*

***P12:****  “It’s really essential for us to have that freedom, because otherwise there would be […] pressure from all sides. I also think that this is truly a key factor for it to work.“*

The necessity of a genuine value proposition emerged as a recurring theme to overcome implementation barriers throughout the workshop discussion, and was perhaps most succinctly captured in the following contribution:

***P15:****“As providers, we need to be clear about how exactly patients benefit from the app, how we as providers benefit, what we believe is actually positive for the patients—what the [patient] might also intuitively see. Not just ‘I’ll go along with it,’ but ‘hey, I see this as a tool that’s good for me and my patient, which is why I use it, because it might also save me work in the long run.”*

According to our workshop participants, key barriers further include the lack of integration with hospital IT systems and the absence of open Application Programming Interfaces (APIs). During the ILIA trial, clinicians were required to document any patient contact separately in the digital dashboard and in the electronic health record (e.g., SAP), leading to redundant work and manual data transfers. As highlighted by another workshop participant, digital tools would only integrate sustainably in existing workflows,

***P5:****“ […], if it really becomes a part of everyone’s daily routine, I mean the clinical routine. For example, that everyone has to use it, that it becomes a kind of obligation. […] And I think with digital tools, one person can use it, another can use it, it’s a bit up to each person to find their own benefit, so there needs to be something more standardized.”*

Cost and sustainability concerns emerged as a prominent theme in the workshop discussion. A representative of the digital health industry (P4) emphasized the financial burden associated with digital therapeutics, highlighting licensing costs, the maintenance of digital infrastructures, and the ongoing training required to ensure proficient clinical use as key barriers to long-term institutionalization. Building on this, one participant articulated a more fundamental challenge and stated that the successful implementation of digital tools may ultimately require a rethinking of existing treatment protocols altogether, a transformation that is currently hampered by the absence of coherent, evidence-based implementation guidelines:

***P4****: “ The point is to change the way we treat. And that’s the difficulty […] to change that while you have not a coherent manual in how treatment should be. If you do psychotherapy […] you have a clear manual, therapists follow it, you do weekly supervision, you discuss how did you handle that situation: You streamline it, everyone treats the same way. And the better the manual adherence, the better the outcomes. And here we don’t have that because it’s still in this moment phase.”*

According to participants, a further barrier to the long-term implementation of digital therapeutics lies in the structural separation between inpatient and outpatient care in Germany that might hinder a smooth discharge during the sensitive transition from inpatient to outpatient treatment. According to another workshop participant, adequate discharge management raises the opportunity to integrate digital tools in a targeted manner and overcome this barrier:

***P4:****“ It depends on planning early enough—where will the patient be discharged to? And within the structure, where will they be discharged? And with the senior doctor one already considers if they would be suitable to use ILIA.“*

## Discussion

This qualitative-descriptive implementation analysis of stakeholder experiences during an ongoing multicenter RCT shows both the promise and the complexity of integrating digital therapeutics into psychiatric care. The adopted R(E)-AIM framework proved valuable in structuring insights across relevant domains and identifying leverage points for improved implementation (Table 2).

**Table 2:** Summary of key findings among the R(E)-AIM framework

R(E)-AIM dimension	Observations (ILIA-Study)	Type (Barrier, facilitator)	Recommended (Future) implementation strategies
**Reach**	Among patients meeting the digital eligibility criteria, skepticism towards digital monitoring was rarely the stated reason for declining	**Facilitator**	-Implement regular recruitment initiatives (e.g. newsletter) -Extend recruitment to outside existing outpatient structures (e.g. peer-groups, inpatient settings, living groups)
	Clinician gatekeeping behavior drove variation in recruitment success across sites more than patient factors	**Barrier**	-Develop clear communication strategies on the benefits of digital tools -Provide adequate incentives for clinician engagement -Target patients directly in the recruitment strategies
	Only a small portion of on-boarded clinicians actually recruited patients	**Barrier**	-Incentivize clinicians to meet recruitment targets -Introduce standardized eligibility screening checklists to counteract clinician gatekeeping bias
**Adoption**	Co-design involvement prior to the RCT created a patient-informed app design that matched real-world usage patterns	**Facilitator**	-Institutionalize patient co-design as a design quality standard in future digital health RCT development
	Individual on-boarding for participating clinicians, information material and centralized study support by researchers reduced cognitive, technical and time burden for clinicians	**Facilitator**	-Individual user onboarding as minimum standard; superior to group-only formats -Dedicated digital coordinator role is essential for sustainable blended care and clinical staff should not absorb technical support functions
	Adoption among exemplary patients varied and appeared dependent on internal and external circumstances, including personality traits (e.g., patient activation) and symptom severity	**Facilitator/Barrier**	-Develop onboarding pathways for patients without established self-management skills to build baseline engagement capacity -Implementation strategies should target motivational states and include educational components on the core idea of preventive interventions
	Reduced or absent app engagement (“monitoring silence”) observed as an adoption-level phenomenon and a potential future analytic signal Use of escalation protocol (involvement of emergency contacts) in case of non-adoption	**Facilitator**	-Integrate passive/sensor-based monitoring as a complement to active self-reporting -Build clinical protocols that treat reduced app engagement as a clinically meaningful signal rather than an adoption failure -Create escalation protocol and include backup contact attempts (e.g. relatives) when app engagement drops
	Low and varying clinician engagement/ adoption rate and additional time burden for clinical staff Suboptimal workflow integration and thus burden from interruptions, not total time volume	**Barrier**	-Integration into existing technical workflows of clinicians (no double documentation) -Formalize clinician adoption as part of the clinical role with protected time or reimbursement -Introduce a minimum engagement standard in the study protocol and do not rely on voluntary engagement
**Implementation & maintenance**	Delegating onboarding and app-related tasks to digital support staff reduced clinician burden Successfully integrated emergency contacts when patients unreachable	**Facilitator**	-Focus on human-supported/hybrid interventions -Create extended care teams (nurses, peer support workers, family) with defined roles in future real-world implementations -Social contacts are frequently overlooked implementation facilitators, formally integrate relatives into implementation design
	Integration with existing electronic documentation systems technically challenging Long-term maintenance requires integrated infrastructure	**Barrier**	-Prioritize EHR integration and open API standards in your implementation strategies -Include early financial feasibility analysis in the planned implementation strategies (e.g. integration costs)
	Clinicians will not integrate an additional monitoring task into their workflow without reimbursement or protected time	**Barrier**	-Link clinician engagement to quality indicators or professional development credits to create structural incentive -Distribute responsibilities connected to the digital intervention and create a buffer layer that absorbs administrative and technical tasks

Completeness of screening failures reporting across sites cannot be guaranteed; the associated Reach observations are thus indicative.

Patient engagement with the app varied across the six use cases and appears to be influenced by individual life circumstances, perceived symptom severity, underlying personality traits, and the degree of collaborative use between the patient and clinician. Existing research suggests that personality traits, e.g., neuroticism, can influence health-promoting engagement^26^. Future research should aim to disentangle the relative contribution of individual dispositions, such as personality traits or symptom severity, to the adoption and sustained use of digital mental health interventions. Research suggests that family interventions are a successful psychosocial intervention in reducing the risk of relapse^65^. Studies, such as those by Aref-Adib et al.^9^, further highlight that relatives can play a pivotal role in maintaining engagement with digital interventions by providing ongoing motivation, stability, and practical support. Qualitative findings with patients and professionals support the role of relatives as potential facilitators for intervention delivery^32^. This was echoed by several stakeholders in the ILIA workshop, who emphasized the value of including trusted contacts, especially when the patient may become unreachable. The minority of existing digital mental health applications, however, involve engagement-stimulating social support design elements^8^. Future implementation strategies should evaluate how other stakeholders, such as relatives or peers, can be involved systematically.

Despite thorough planning and adherence to recommended implementation strategies (e.g., on-boarding sessions and training, material support, technical assistance), the adoption by clinical staff remained partly limited. The trade-off between perceived benefit and burden was mentioned as a key barrier to adoption among clinicians during our workshop. This is in line with current studies that identified limited time and capacity for training and adoption as one of the most relevant implementation barriers to long-term adoption^9,10^. Our intervention employs a remote monitoring approach combined with blended care components, which potentially adds complexity to implementation, as both clinicians and patients are required to use the applications continuously. Our workshop discussions illustrated a potential mismatch between top-down implementation strategies and bottom-up clinical realities and reinforced the importance of co-design approaches, which actively involve patients, but also clinicians, in shaping digital tools and their workflows. Adequate education and training can further ensure that clinicians use applications efficiently^21,29,31,63^, and financial incentives or institutional recognition may enhance their sustained engagement^21,29^. Integrating those innovations into clinical guidelines can also help to normalize digital tools as part of routine mental health care^29^. Digital tools are, however, most likely to be adopted when they are seamlessly embedded into existing clinical workflows. In the ILIA trial, clinicians concluded that parallel systems that require additional login, double documentation, or data interpretation will lead to resistance. Such ‘double documentation’ has previously been identified as a major barrier to implementation and emphasizes the need for interoperability between digital tools and existing local clinical infrastructure^21,25^. To date, the majority of hospital information systems are proprietary and closed, with little means to provide interoperability, such as loading data from external apps and sources. In the near future, this situation should change in Europe with the introduction of the European Health Data Space (EHDS) Regulation (EU) 2025/327, which defines requirements for data exchange standards in medical information systems^66^.

During the ILIA study, we delegated technical support to the study team, in line with previous implementation research that emphasizes the value of centralized technical support^22,64^. However, this raises a broader question of transferability. The strategies deployed, i.e., structured onboarding, on-demand technical assistance, and protocol-driven follow-up in cases of non-use, were feasible because they were funded and staffed as part of an RCT, resources that are rarely available in public-sector services. It is therefore useful to distinguish strategies that are largely context-independent, such as interoperability with hospital information systems or reimbursement pathways, from those that are artefacts of trial infrastructure. Expanding clinical capacity through support staff was identified as a promising strategy in recent research^30,64^. Shifting technical and monitoring tasks to study nurses or digital coordinators can, for instance, ensure that clinical staff are not overburdened with administrative or technical responsibilities^64^. Further, so-called “digital navigators” can mentor patients or help aggregate data for efficient clinical use, thereby easing successful implementation^64^. While such roles may be viable in university hospitals or academic settings, their affordability and effectiveness in community-based or non-academic outpatient settings remain largely untested. This reflects a fundamental tension between implementation strategies feasible within the structured ecosystem of a clinical trial and those achievable in routine psychiatric care.

Sustaining the use of digital therapeutics beyond the research context remains a significant challenge. Key sustainability factors, such as cost-effectiveness, stable funding, and integration into existing infrastructure, are often not sufficiently considered during the early stages of development^10^. Digital therapeutics often require licensing, the creation and continuous maintenance of costly digital infrastructures, as well as ongoing training to develop necessary digital skills that ensure proficient use of the tools^21,25^. It also underscores the central role of financial sustainability in achieving lasting implementation. Smith et al.^30^ highlight that early analysis of financial or operational feasibility is crucial for long-term implementation. Digital therapeutics must be evaluated for how they can be seamlessly integrated into existing workflows in pre-conceptualization processes^25^. Overall, our findings confirm the previously well-documented challenges in the implementation of digital interventions in mental health care and extend them by demonstrating a prospective, iterative approach in which implementation considerations are integrated throughout an ongoing multi-site RCT^55^. We engaged interdisciplinary stakeholders to discuss and refine multiple barriers, facilitators, and implementation strategies in real time, while accounting for both patient and clinician perspectives within routine psychiatric care.

This implementation analysis is subject to several limitations. First, the findings are based on a single workshop with a limited and potentially self-selecting group of participants. Consequently, the results may be subject to selection bias. While efforts were made to include diverse stakeholder perspectives, including clinicians, researchers, and digital health developers, participation remained lower than anticipated, particularly among frontline clinicians. Of the 80 clinicians invited, six frontline clinicians attended (a participation rate of approximately 7.5%), in addition to the two principal investigators, who are themselves clinicians. The perspectives of non-participating clinicians, plausibly the most informative group with respect to why adoption did not occur, are therefore underrepresented in our data. This constitutes a directional bias favoring already-engaged stakeholders. Patients with lived experience, as the primary end-users of the digital monitoring system, were under-represented in this workshop, as they were not directly included in the participatory session but instead contributed through three separate pre-RCT focus groups. However, it should be noted that the primary focus of this workshop was on identifying implementation barriers, whereas individuals with lived experience had been involved in focus groups during earlier developmental stages. Patients´ perspectives on app usability and fit with daily life and personal recovery goals may differ substantially from those of clinical staff and coordinators. However, we also plan to evaluate patients’ experiences with the app and its functionality post-RCT using qualitative interviews, as well as dyadic analyses between clinicians and patients with lived experience, to assess implementation and changes throughout the treatment period. Beyond the financial relationships reported in the Competing Interests statement, a potential for interpretive bias should be acknowledged as three co-authors are employees and/or shareholders of the company providing the digital intervention and participated in the stakeholder workshop. As procedural safeguards, the qualitative coding, synthesis, and interpretation of the workshop data were performed exclusively by academic researchers without company affiliation, and the workshop was moderated by an academic research associate. Nevertheless, the participation of stakeholders with a commercial interest in the intervention may have influenced the framing of barriers and facilitators during the workshop discussion. Next, the analysis reflects a mid-study, cross-sectional snapshot of implementation processes during the active trial phase, meaning that long-term sustainability and integration of the digital intervention into routine care were not assessed. Findings are derived from a specific national healthcare context, i.e., within Germany. Transferability to settings with different regulatory environments, reimbursement structures, or digital infrastructure should not be assumed. Readers are encouraged to assess the degree of contextual similarity before applying these findings. Third, the data collection was qualitative-descriptive and exploratory in nature. While we applied structured frameworks (e.g., RE-AIM), no formal quantitative evaluation of the Effectiveness category on clinical outcomes was conducted at this stage. The workshop was intentionally designed as a low-threshold, pragmatic-oriented reflection format, which prioritized contextual insights over comprehensive qualitative coding. While the participatory real-time categorization by all 16 stakeholders, the triangulation of codes with the participant-generated worksheet data, and the resolution of ambiguities through participant consultation provide validation mechanisms appropriate to this framework-guided, exploratory design, the directed content analysis of the workshop transcript was performed by a single researcher. Above, although use case selection was anchored in objective usage metrics and completed prior to the workshop, the choice of use cases within each usage pattern involved researcher judgment. Lastly, the top-down implementation strategy used in the ILIA study (i.e., study-led onboarding, centralized coordination, controlled environment) may have lacked sufficient opportunities for co-creation and local adaptation. Implementation science increasingly recognizes the value of participatory approaches, in which clinicians are not only trained, but also actively involved in shaping interventions. Future studies should therefore explore more collaborative, bottom-up implementation models that foster ownership and adaptability.

The above-mentioned selection bias has some implications for the interpretation of our findings. Because the workshop over-represented engaged clinicians, the barriers identified are predominantly those visible to stakeholders who had already committed to the intervention. This included structural barriers such as workflow integration, onboarding procedures, alert configuration, lack of time, and communication pathways. More fundamental forms of clinician disengagement, such as skepticism regarding the clinical value of digital symptom monitoring, perceived threats to clinical autonomy, or a general lack of interest in digital interventions, might be underrepresented, as clinicians holding such views were unlikely to attend. Accordingly, the implementation strategies derived from this workshop should be understood as addressing necessary conditions for broader clinician adoption. Strategies targeting fundamentally disengaged clinicians require dedicated studies, for example through targeted non-responder outreach.

In conclusion, real-world success of digital therapeutics for people with SMI may depend less on technological sophistication than on organizational structure, perceived benefit in clinical decision-making, and relevance for those affected. In line with prior work in the field^10,21^, we conclude that digital applications for mental health, particularly for SMI, cannot be effectively implemented without appropriate human support. The platform design used in the ILIA study addresses the well-researched importance of human-supported digital interventions. Each patient account is connected to an account of the treating clinician who receives alerts in both scenarios, in response to critical symptom threshold exceedance and in cases of non-use^54^. According to the ILIA protocol, clinicians and patients are both expected to proactively reach out to each other in case of an alert period, thereby reinforcing the shared responsibility. Study staff additionally supported monitoring and reached out to patients and clinicians in the case of lacking engagement according to the protocol. The ILIA study thus represents an effort to embed human-supported and collaborative care structures into digital symptom monitoring. While this support may come from study personnel in controlled research settings such as RCTs, involvement of clinical staff, digital health coaches, peer supporters, or other professionals is crucial in real-world contexts. In the ILIA study, further qualitative evaluations are planned to deepen the understanding of implementation dynamics^54^. This will include semi-structured interviews with participants from the intervention group, as well as audio recordings of clinician–patient conversations. These data sources are expected to provide valuable insights into how users perceive and engage with digital relapse prevention in real-world settings and may further help to refine the ILIA system for broader implementation in routine care. The strategies discussed here relate primarily to onboarding and early adoption rather than to long-term adherence, which continues to pose a challenge in digital mental health research. Distinguishing the factors underlying short-term uptake from those required for sustained engagement over several years remains an ongoing area for future investigation.

## Methods

### Study background and design

**Fig. 9:**
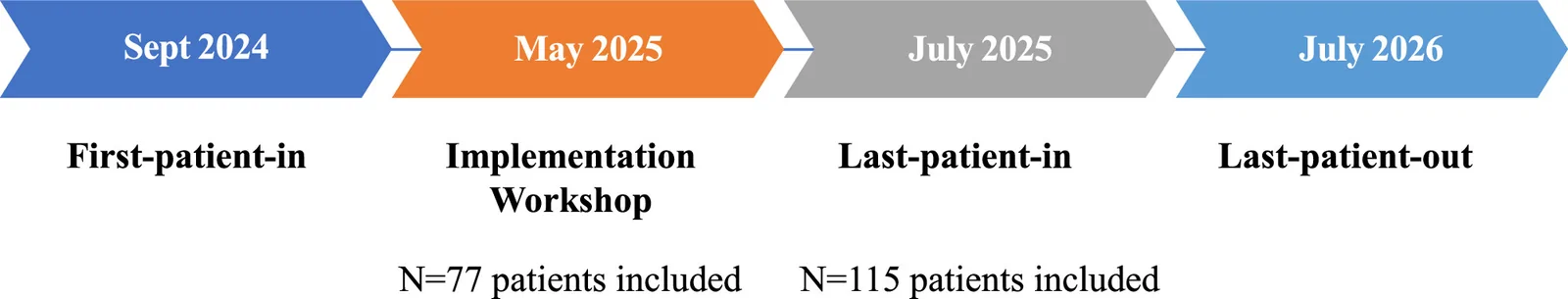
Key milestones of the ILIA study timeline from first patient enrollment to study completion. The first patient was enrolled in September 2024, followed by an implementation workshop in May 2025 (*N* = 77 patients included at that point). Recruitment closed in July 2025 with a final sample of 115 patients enrolled, exceeding the predefined recruitment target of 110 participants. Data collection was completed in July 2026.

The ILIA trial is a multicenter RCT evaluating the effectiveness of an app-based relapse monitoring in outpatients with schizophrenia and schizoaffective disorder (ICD-10 F20/F25)^54^. The digital intervention is delivered in addition to treatment as usual (TAU) and includes weekly regular mobile self-report assessments. The app triggers automated alerts to both patients and clinicians when increased relapse risk is detected by a built-in algorithm^54^. Patients receive in-app visual feedback on their relapse risk, and clinicians can monitor the course of relapse risk in a web-based clinic portal. The digital intervention focuses on shared decision-making in post-alert clinician-patient communication. The study duration is 12 months and includes 3-monthly quantitative assessments on various outcome measures. The primary outcome is the reduction in psychiatric re-hospitalizations in the intervention group compared to the TAU-only group. The first patient was enrolled in September 2024 and recruitment ended in July 2025. We exceeded the predefined target sample size of 110 participants with 115 patients enrolled in the study (Fig. 9). Further details can be found in the published study protocol^54^ and on the German digital registry of clinical trials (DRKS-ID: DRKS00034991).

The ILIA study was conducted in accordance with the Declaration of Helsinki. The ILIA study is approved by the review board of the Ethics Committee of the Technical University of Munich (approval number: 2024-242-S-KK). The ethics committees of all participating clinical sites adopted this lead opinion and approved the study, including Ethics Committee of the LMU Klinikum München (approval number: 24-1062), the kbo-Isar-Amper-Klinikum Region München (approval number: mb24046), the Bezirkskrankenhaus Augsburg (approval number: 25-0175). The Ethics Committee of the Charité Universitätsmedizin did not issue a separate ethics approval, but adopted the lead opinion of the ethics committee of the Technical University of Munich. The Clinical Trial Office (CTO) of the Charité Universitätsmedizin Berlin provided regulatory guidance and collaborated with the study team in finalizing a data protection concept for the Berlin site.

This study is defined as a qualitative-descriptive implementation evaluation embedded within an ongoing multicenter RCT, i.e., a Type 1 hybrid effectiveness–implementation trial^55^. In such trials, effectiveness outcomes are prioritized, while implementation data are collected prospectively and in parallel to inform future scale-up^55^. Our methodological approach followed a pragmatic, qualitative-descriptive epistemological stance^56,57^, seeking to characterize implementation dynamics across heterogeneous clinical settings, rather than test predefined causal hypotheses. To support the successful roll-out of the ILIA study, we introduced various structured implementation strategies and proactively addressed common implementation barriers such as top-down designed interventions, limited familiarity, unclear clinical responsibilities or technical uncertainty and thereby aimed to lower the threshold for patient and clinician engagement during the study.

### Data collection during the participatory workshop

The stakeholder workshop served as the primary qualitative data collection method. Participants were recruited using purposive sampling to ensure representation across key stakeholder groups directly involved in trial delivery. We invited clinicians who had been in contact with the ILIA study team at least once since study initiation (*n *= 80) via E-Mail, including clinicians that already recruited participants, as well as those who only reported screening failures. Those invitations also included clinical site leads (*n *= 8). In addition, members of the ILIA study team were invited, including principal investigators (*n *= 2), research associates (*n *= 3), and student assistants (*n *= 3). Representatives from the digital therapeutics company, including UX-, technical-, and strategic staff, were also invited (*n *= 5). All workshop participants provided written informed consent prior to participation, including consent for audio recording, pseudonymized transcription, and publication of the results.

The RE-AIM (Reach, Effectiveness, Adoption, Implementation, Maintenance) framework has been widely applied in implementation science^41,48,50^ and, more recently, also in digital health contexts^49,52^. The analysis was guided by the updated RE-AIM framework^49^, which incorporates refinements for contemporary implementation contexts, including digital health interventions. It has demonstrated its value in capturing implementation-relevant dimensions, often underrepresented in traditional trial designs.

The workshops´ primary objective was to analyze implementation experiences along four of the five R(E)-AIM dimensions: Reach, Adoption, Implementation, Maintenance. Since we followed a qualitative-descriptive study design and collected cross-sectional data within an ongoing RCT, we did not include any quantitative interim assessments in the Effectiveness domain. Effectiveness outcomes will be reported upon trial completion. We therefore refer to the adopted R(E)-AIM framework throughout this article. The pragmatic application of a subset of RE-AIM dimensions is consistent with the updated RE-AIM guidance, which states that studies should address those RE-AIM components most appropriate for their question, setting, and stage of research^49^. The adopted framework was introduced to all participants prior to the group discussion. We used a structured worksheet aligned with the Reach, Adoption, Implementation and Maintenance categories, to guide real-time discussion, brainstorming, and prioritization of key implementation aspects. We selected RE-AIM rather than a determinant framework such as CFIR because the two frameworks serve different functions^67^. CFIR specifies factors influencing implementation, whereas RE-AIM structures implementation outcomes. As this study is an outcome-oriented mid-trial implementation evaluation, RE-AIM provided the appropriate scaffold, particularly for the Reach and Maintenance dimensions, which have no counterpart in CFIR. Determinant-level insights were nevertheless captured as barriers and facilitators coded within each domain.

Reach was conceptualized in terms of the recruitment and engagement of both patients and clinicians. The moderator thus started the workshop with an update on recruiting numbers, drop-outs and reach of clinicians. Our exploratory analysis focused on identifying which patients (sub-) groups were reached and examining clinician participation and contribution to recruitment. Adoption was discussed in terms of applied implementation strategies and exemplary patient use cases. The cases provide valuable contextual information on how the intervention was adopted within real-world clinical settings. The use cases were selected from the eight participating sites using maximum variation sampling^68,69^. The candidate pool for use case selection comprised all patients randomized to the intervention arm in whom the app had been installed at the time of selection. The six use cases were selected in preparation for the workshop (26 May 2025), at which point 77 patients had been enrolled overall, 39 of whom had been randomized to the intervention arm. The use cases were drawn from this intervention-arm pool. Prior to the workshop, SH, LE, and DJ screened the objective app usage and portal data of those 39 patients (data completeness, alert periods, documented post-alert contacts, inactivity intervals) together with basic clinical and site information. Recurrent usage patterns were identified across the cohort, and for each pattern one exemplary case was selected by team consensus to maximize variation across three major dimensions identified as relevant to digital mental health implementation: (1) patient adoption rate at trial midpoint, (2) patient characteristics, and (3) local clinical dynamics/adoption of the respective clinician. The cases are not presented as representative of all trial sites, but as purposively selected illustrations of distinct implementation dynamics^68,69^. Selection was anchored in the observable usage metrics and was completed before the workshop, i.e., prior to and independent of the qualitative data collection. The cases are presented as purposively selected illustrations of distinct implementation dynamics, not as representative of the full intervention arm. The use cases were discussed along semi-structured impulse questions provided on the worksheet (Supplementary Information 1). Implementation focused on how the intervention was integrated into existing workflows, and discussions were based on results found in the previous R(E)-AIM dimensions. Maintenance was understood as sustainable long-term implementation and included open discussions on potential scenarios for future implementation strategies.

For all R(E)-AIM dimensions, participants collaboratively mapped their experiences in real time onto the provided worksheet. Notes were either collected collaboratively via a shared online document for people who participated online, or on print-versions for participants on-site (Supplementary Information 1). This process was consensus-based and facilitated by the workshop moderator to ensure structured engagement across all dimensions. All 16 workshop participants contributed to the qualitative data through the collaboratively completed worksheets and the moderated group discussion. The approach aligns with previous RE-AIM-informed implementation evaluations using stakeholder-informed qualitative synthesis^48,50^ and allowed for a context-sensitive triangulation of real-world experiences. Additionally, the workshop was audio recorded.

### Data analysis

Research on the use of the RE-AIM framework emphasizes the need to move beyond descriptive application of the framework toward contextual analysis. This includes understanding which barriers or facilitators emerged in a given implementation context^49,51^. The use of the implementation worksheet during the workshop represented a form of simultaneous data generation and real-time collaborative categorization, as the worksheet was structured a priori along the four R(E)-AIM domains, and all 16 stakeholders collaboratively and consensually assigned their contributions to these domains in real time. The initial domain-level categorization was thus performed participatorily by the stakeholders themselves during data generation. The four domains, together with their definitions, constituted the deductive top level of the coding frame and were fixed prior to analysis. Within this overarching R(E)-AIM structure, barriers to implementation were additionally categorized by participants in real time along five predefined levels (patient, clinician/professional, structural, technical, and communication/information) during an initial worksheet task, such that both the R(E)-AIM domains and this nested level-based categorization of barriers were generated participatorily during data collection.

The workshop audio recording was transcribed verbatim by a member of our research team and pseudonymized during transcription. The transcript was then analyzed by one researcher (SH) using directed content analysis^58^, an approach in which existing theories or empirical frameworks guide the development of initial coding categories. The four deductive R(E)-AIM domain codes were applied, taking the participants’ real-time worksheet placement as the initial domain assignment, and sub-categories of barriers, facilitators, and implementation strategies were derived inductively within each domain. Full transcripts were not returned to participants; however, where ambiguities arose during coding, the respective workshop participants were consulted to clarify their contributions. The full coding frame (codebook), including domain and sub-category definitions, anchor examples, and coding rules, is provided in Supplementary Information 2. All transcript codes were systematically cross-checked against the participant-generated worksheet data and the worksheet entries were merged into the category system. No statistical inter-coder reliability coefficient was calculated, as such coefficients presuppose independent parallel coding by multiple coders. In the present design, the initial domain-level categorization was performed collaboratively by the participants themselves. To mitigate the potential for interpretive bias, the qualitative coding, synthesis, and interpretation were performed exclusively by academic researchers without commercial affiliation, and coding was triangulated against the participant-generated worksheet data. No coding software was used. Transcript were transcribed and coded manually. Reporting of the qualitative component follows the COREQ checklist^70^ (Supplementary Information 3).

## Supplementary information

Supplementary Information 1: Worksheet of the Implementation Workshop

Supplementary Information 2: Codebook

Supplementary Information 3: COREQ-Checklist

## Data Availability

The datasets generated in this study are not publicly available. Under the study's ethics approval, only anonymized data may be released, and the qualitative workshop data cannot be shared even in pseudonymized form, as a group-discussion transcript cannot be fully anonymized without residual re-identification risk. The data also represent a mid-study snapshot collected prior to completion of the primary randomized controlled trial. The anonymized quantitative dataset needed to interpret the reported baseline and usage findings will be made available alongside the primary RCT publication. Until then, requests can be directed to the corresponding author, S.H. (selina.hiller@tum.de). Access will be granted to qualified researchers for non-commercial use after a signed data transfer agreement and within the constraints of the ethics approval. Individual data will be retained for 5 years after the end of the ILIA study (31 December 2026) and deleted thereafter, i.e., by 31 December 2031.

## Code availability

Statistical analyses were performed using R (version 4.4.1) and RStudio. The analyses consisted of descriptive statistics only, and no custom code or scripts were generated.
