# Supplementary material for: Implementation strategies for digital relapse prevention in schizophrenia identified during a randomized controlled trial
Source: NPJ Digit Med. 2026 Oct 30;9:827. doi: 10.1038/s41746-026-03435-2 (PMC13722681; doi:10.1038/s41746-026-03435-2)
Supplement: Supplementary file 1 [file 41746_2026_3435_MOESM1_ESM.pdf]

ILIA-Implementation Workshop

“Co-Designing digital relapse prevention”

Worksheet

Date: 26.05.2025, 14:30

Participants:

Task 1: Barrier Map for Previous Study Implementation

Processing time 15 minutes, one pen color per person, 15 minutes discussion

Category	Implementation Barriers
**Patienten level**	
**Clinician / professional level**	
**Structural level**	
**Technical level**	
**Communication and information**	

Task 2: Guiding Questions for the Discussion of Use Cases (approx. 90 min)

Use Case 1: Patient in Continuous Alarm

📲 App Functionality & Further Development

- Which functions could be helpful in the long term if the values remain permanently in the critical range?
- What options are there to support patients in self-assessment?

Use Case 2: Irregular Questionnaire Completion, Patient Never Reached, Patient Admitted Inpatient

📲 App Functionality & Further Development

- How could questionnaire completion have been better ensured?

🤝 Commitment & Continuity of Care

- How frequently should the study team contact the patient?
- How realistic is this close contact in real-life settings?
- How should an increase in manic symptoms in F25 diagnoses be assessed in relation to a relapse?

Use Case 3: Very Intensive Use of App Functions by Patient

🤝 Commitment & Continuity of Care

- What could be the reasons that this patient uses the app particularly reliably and thoroughly?

📲 App Functionality & Further Development

- How can the digital application help to channel high-frequency contact needs into structured, orderly processes?

Use Case 4: Irregular Completion (“Forgot because I feel fine”)

🤝 Commitment & Continuity of Care

- How can patients be encouraged to use the app even when they feel well?
- Why is the app forgotten despite push notifications and study monitoring?

📲 App Functionality & Further Development

- Why was the second alarm triggered? Everything seemed to be in the green range. It is marked red, but the system did not generate a notification?

Use Case 5: Patient Suddenly Unreachable, Learned About Hospitalization of a Family Member, Now Back in Outpatient Care

🤝 Commitment & Continuity of Care

- How could a faster/better response have been possible?
- We were able to conduct the next assessment with the patient despite home-treatment (German: stationäquivalente Behandlung, StäB), however, the clinician hasn't seen the patient since February. How should we handle their assessment (postpone, skip, etc.)?

📲 App Functionality & Further Development

- Why was no alarm triggered? Because there was only a very slight deterioration, never enough to reach the threshold?

Use Case 6: No Follow-up Appointments Attended, Chronic Pain Patient, Symptom Reporting Always Linked to Pain Symptoms

🤝 Commitment & Continuity of Care

- What could help ensure that patients remain in monitoring even with repeated appointment cancellations?
- How can contact with patients be maintained if in-person appointments do not take place (e.g., peer support, digital consultation)?

📲 App Functionality & Further Development

- How can a digital application respond meaningfully to comorbidities (e.g., chronic pain), and to what extent should the app address different symptom sources?
- How can the digital application help to channel high-frequency contact needs into structured, orderly processes?

Task 3: Discussion along the RE-AIM framework

Processing time **30min**, one pen colour per person, 15min discussion

Dimension	Guiding Questions	Discussion
**Reach**	Who was reached? For which patients is the app particularly suitable?	
	Which target groups were left out (e.g., due to digital barriers, language, cognitive load)?	
**Effectiveness**	[not a primary focus in the workshop]	
**Adoption**	[based on the use case analysis] How is the app accepted by healthcare providers and teams?	
	[based on the use case analysis] Which factors promote or hinder usage?	
**Implementation**	How well can the app be integrated into existing workflows?	
	Which people and organizational levels need to be involved in implementation processes?	

	What support do healthcare providers need for implementing new interventions/workflows, etc.?	
**Maintenance**	What is required to ensure monitoring remains a part of routine care?	
	Who should take responsibility for the app in the future (promoting the app, setting it up, monitoring, contacting users)?	
**Acceptance**	How do patients and healthcare providers assess usability, effort, and benefit?	
