# Supplementary material for: Implementation strategies for digital relapse prevention in schizophrenia identified during a randomized controlled trial
Source: NPJ Digit Med. 2026 Oct 30;9:827. doi: 10.1038/s41746-026-03435-2 (PMC13722681; doi:10.1038/s41746-026-03435-2)
Supplement: Supplementary file 2 [file 41746_2026_3435_MOESM2_ESM.pdf]

Supplementary Information 2. Coding frame (codebook) of the directed content analysis along the adopted R(E)-AIM framework

Development of the coding frame. The deductive top level (Level 1) of the coding frame consisted of the four R(E)-AIM domains (Reach, Adoption, Implementation, Maintenance), operationalized as defined in the table. These domains and their definitions were fixed a priori: they structured the workshop worksheet (Supplementary Information 1, Task 3) and served as the categories of the real-time collaborative categorization performed by all 16 participants during the workshop (simultaneous data generation and participatory categorization). Within the overarching R(E)-AIM structure, implementation barriers were additionally categorized by participants in real time along five predefined levels (patient, clinician/professional, structural, technical, and communication/information) during an initial worksheet task (Supplementary Information 1, Task 1). These levels form a deductive sub-axis for barrier sub-categories and are recorded in the dedicated column. Sub-categories (Level 2) were derived inductively from the verbatim transcript and the participant-generated worksheet data during the subsequent directed content analysis, performed by one researcher (SH), and were classified as barriers, facilitators, or implementation strategies within each domain.

Coding rules.

- Unit of analysis: meaning units (statements or coherent passages addressing one implementation aspect). A unit could be assigned to more than one domain if it addressed several dimensions.
- Validation: all transcript codes were systematically cross-checked against the participant-generated worksheet data, which were merged into the category system.
- Workshop language was a mixture of German and English, therefore Anchor examples can be in both languages.

Level 1: R(E)-AIM domain (deductive)	Domain definition (as operationalized in this study)	Type	Barrier level (deductive sub-category)	Level 2: sub-category (inductive)	Sub-category definition / coding rule	Anchor example (if applicable)
**Reach**	Recruitment and engagement of both patients and clinicians: which patients/sub-groups were reached and screened, reasons for non-participation, and clinician participation in recruitment.	Barrier	Patient level	**One-year study duration deters participation**	Statements that the 12-month commitment discouraged patients from enrolling, independent of the app itself.	*many patients didn't want to enter the study because they said like, one year, I don't know what I will be doing in one year. Maybe I won't be living anymore in Berlin and then I can't participate (line 200 – 202)*
		Barrier → Strategy	Patient level	**Insufficient financial compensation**	Reports that the reimbursement level, or its distribution across visits, was perceived as too low. Raise or	*in ILIA bekomme ich ja, selbst wenn ich nicht die App habe, bekomme ich ja noch viel mehr ärztliche Visiten gespricht Also*

Level 1: R(E)-AIM domain (deductive)	Domain definition (as operationalized in this study)	Type	Barrier level (deductive sub-category)	Level 2: sub-category (inductive)	Sub-category definition / coding rule	Anchor example (if applicable)
					redistribute compensation (e.g. more for the first/last visit) to improve reach and participation.	*ich habe eine engmaschigere Versorgung und das war dann gar nicht erforderlich dass da noch extra viel Geld fließt (line 283-285)*
		Barrier	Patient/ Structural level	**Eligibility and screening constraints**	Eligible-appearing patients excluded through relapse-window, language, competing-study, adherence criteria at screening, exacerbated symptoms, or skeptics towards smartphones.	*Some diagnoses, which were often "Drogeninduzierte Psychose" they weren't included. And a few of them because of the language, because we have the Vietnam ambulance and the Arabic ambulance and so they don't fulfill the criteria of speaking German (line 103-106)*
		Facilitator	–	**Broadened, clinically-judged inclusion criteria**	Loosening of criteria (up to markedly ill, depot patients, flexible relapse definition) to widen who could be reached.	
		Facilitator	-	**Recruitment of inpatients before discharge**	Encouragement to recruit patients in their last inpatient weeks, when relapse risk (and thus benefit) is highest.	*I want to remind you that patients who just come from the inpatient are also very welcome. Actually. They can already be included in the study in their last one or two weeks of inpatient stay, which should make it easier actually, that they stay in the study (line 72-75)*
**Adoption**	Uptake and use of the intervention by patients and clinicians in real-world settings, examined largely through six purposively selected patient use cases and discussion of usage patterns.	Barrier	Patient level	**Misunderstanding of the change-based rating concept**	App outputs distorted because patients rated absolute symptoms rather than week-to-week change, producing continuous alerts	*A patient in continuous alert had not grasped that items ask about change vs. the previous week rather than symptom level. (transcript, P6, Use Case 1)*
		Barrier	Patient level	**Symptom-driven / relapse-driven disengagement**	App use ceasing precisely around symptom exacerbation or relapse, lack of data when it would be most informative	*das Traurige in dem Sinne dass die wichtigsten Daten ja fehlen. Also es ist ja genau diese spannende Zeitperiode, nämlich kurz vor einem Rückfall, ist natürlich, auch prädestiniert bei Patienten dafür, nicht mehr auszufüllen (line 844-845)*
		Barrier	Patient level	**Disengagement in low-symptom phases**	Reduced completion when patients feel well ('forgot because I feel fine'), or were occupied with other tasks.	*Sometimes they forget and then do it again, and then we contact them and it was just like, oh yeah, I forgot, but this week I used it again. Or like, oh yeah, I had my exams. I was like. Super super stressful, but now I use it again (line 580-582)*

Level 1: R(E)-AIM domain (deductive)	Domain definition (as operationalized in this study)	Type	Barrier level (deductive sub-category)	Level 2: sub-category (inductive)	Sub-category definition / coding rule	Anchor example (if applicable)
		Barrier	Patient level	**Somatic comorbidity distorting symptom signals**	Deteriorations reflecting somatic conditions (e.g., chronic pain) rather than psychiatric worsening, complicating interpretation and making more objective indicators important	*Es war davor keine Verschlechterung, sondern es ging wieder hoch, weil er eine Woche stabil war und dann hat er, und das hat er selber als Live-Event reingeschrieben, die Nachricht bekommen, dass sein Hals versteift werden muss. Und dann ging sofort halt das runter (line 1145-147)*
		Barrier	Technical level	**Below-threshold deterioration not triggering alerts**	Clinically relevant worsening remaining under the alert threshold, so no notification is generated	*A patient reported feeling worse weekly, yet no alert was ever triggered because deterioration never reached threshold. (transcript, Mohammed/P3, Use Case 5)*
		Facilitator	Patient level	**Intrinsic motivation and illness self-management**	Sustained, thorough use linked to activation, self-education about the illness, and strong reassurance-seeking behavior	*die Patientin ist sehr sozial aktiv im Rahmen ihrer chronischen Erkrankung. Sie bildet sich privat über die Erkrankung weiter, nimmt an diversen Kursen teil, pflegt lange Freundschaften und ist halt generell einfach eine Patientin mit einem sehr, sehr guten Rückversicherungsverhalten (line 960-963)*
		Strategy	–	**Onboarding, psychoeducation and transparency on the change monitoring concept**	Enhanced onboarding/leaflets and early-warning-sign education to convey how self-assessment should be interpreted.	*the doctor should be able to do or in our case the study staff should be able to do is to explain to the patients the change because this is how the drug works right like we want them to understand the principle of change and then we are just asking about that (line 762-764)*
		Strategy	–	**Patient-adjustable notifications and app customization**	Allowing patients to configure notifications/thresholds so the tool fits diverse usage styles without clinician burden.	*Some patients find it very intriguing if there was a daily pop-up on the smartphone but this specific person said, he was like, I would like to have a pop-up every day because I'm forgetting things so easily. (line 491-493)*
		Strategy	–	**Non-completion as a predictor of relapse**	Treating cessation of app use as an analytic warning signal rather than mere missing data.	*consider putting no show as a predictor (line 893)*
**Implementation**	Integration of the intervention into existing clinical workflows and structures: documentation load, staffing/time, portal handling,	Barrier	Clinician level	**Clinician workload and lack of dedicated time, limited workflow alignment**	Rating and study tasks competing with routine clinical duties; incentive perceived as too low for non-salaried clinicians.	*für die meisten Ärzte ist das jetzt erstmal ein, sie müssen was von Top drauf machen. Und dafür ist der Incentive einfach zu gering. Wenn ein Stellenanteil ist, dann ist das ja der Job, den du machen musst. Jetzt*

Level 1: R(E)-AIM domain (deductive)	Domain definition (as operationalized in this study)	Type	Barrier level (deductive sub-category)	Level 2: sub-category (inductive)	Sub-category definition / coding rule	Anchor example (if applicable)
	alert-response pathways, and clinician engagement.					*machen die Motiviertesten mit, die an der Forschung interessiert sind. Und das meine ich, das wussten wir ja, das haben wir schon mal diskutiert, kein neues Thema. Ich würde sagen, bei der nächsten Studie müsste man überall mindestens eine 10-20% Arztstelle haben, sich ganz darauf fokussiert (line 411-416*)
		Barrier	Technical level	**Double documentation across systems and lack of APIs**	Having to enter the same information into both the hospital system (e.g., SAP) and the ILIA portal, adding administrative burden.<br>Absence of an open API/protocol to transfer data between the portal and heterogeneous, changing hospital systems.	*wir haben ja diese SAP-Dokumentation und jeder ILIA-Termin muss sehr ausführlich dokumentiert werden. Das heißt... P15 geht sowieso schon nach jedem normalen Termin sofort in SAP rein und schreibt das runter, weil es auch am besten sozusagen gleich nach dem Termin geschehen soll. Und dann muss er halt nochmal entweder gleich danach in ILIA rein, nochmal das gleiche reinschreiben (line 476 – 480)*
		Barrier	Structural level	**Regulatory limits on automated escalation**	Legal constraints preventing the system from directly triggering a treatment step (e.g., routing patients to staff), limiting direct-referral features	
		Barrier	Structural level	**Heterogeneous, site-specific care structures**	Differences in how sites integrate the intervention and control condition, producing variable TAU and implementation across sites.	*dass ein Punkt, den ich mir vorstellen kann, den man auch für zukünftige Studien überlegen müsste, ist, dass man die Tau-, die Kontrollgruppe so ähnlich wie möglich gestaltet und nicht, dass eigentlich eine mögliche doch mehr Versorgung kommt als der übliche Weg (line 318-320)*
		Facilitator	Structural level	**Study team as central coordination and support**	The study team handling onboarding, appointments, follow-ups and acting as help desk to smooth implementation.	*the whole organizational things were like done by our study team. So we were like the help hotline for technical issues. We coordinate all the appointments, all the follow up assessments. So this is what we've done in ILIA to make the implementation as smooth as possible (line 140-142)*
		Strategy	–	**Dedicated digital navigators / study staff / nurse role at sites, involvement of family members**	Proposals for a part-time study nurse, funded clinician or digital navigators who have time to review the portal and offload clinicians.	*I think it would be much more meaningful in the long run, you have one study nurse, half a position at each hospital, and they just go through it the whole time and check your*

Level 1: R(E)-AIM domain (deductive)	Domain definition (as operationalized in this study)	Type	Barrier level (deductive sub-category)	Level 2: sub-category (inductive)	Sub-category definition / coding rule	Anchor example (if applicable)
						patients, your patients, your patients, and then they contact them, so then the physician's load gets less. (line 701-706)
		Barrier → Strategy	Clinician level	**Clinician resistance, limited digital affinity and clear clinical benefit**	Reservations toward digital monitoring on the clinician side (control concerns, transparent patient, benefit). Communicating concretely how the app benefits clinicians and patients so clinicians adopt it as a useful tool.	*ganz klar setzen müssen inwiefern die Patienten genau von der App profitieren, wie der Behandler davon profitiert, was wir glauben, was Positives für die Patienten eben passiert. (line 443-445)*
**Maintenance**	Sustainability of the intervention beyond the trial: sustained engagement over time, continuity across care transitions, and responsibility for and financing of routine-care implementation. This category is closely aligned with the Implementation domain and includes all previously discussed barriers and strategies needed to overcome for long-term implementation.	Barrier → Strategy	Structural level	**Fragmented inpatient–outpatient care transitions → Continuity of care and embedding the app in discharge / relapse-prevention routines**	The German inpatient/outpatient divide impeding continuity, hand-over, and sustained app use across settings. Introducing the app during the last inpatient week within existing relapse-prevention and discharge management to ensure a continuity of care	*dass einfach eben die Versorgung der Patienten einfach verbessert, wird dadurch, durch den Kontakt und durch die Kontinuität einfach auch, dass da immer irgendwie so eine Kontinuität in der Versorgung da ist. (line 1245-1247)*
		Barrier	Structural / clinician level	**Rethinking clinical workflows; Reimbursement and implementation costs**	Concern that restructuring workflows needs also financial backup and top-down decision making	*dass es ja irgendwie super schwer ist, Menschen in neue Handlungsabläufe zu bringen, in neue Routinen und so. Und, dass das meistens meiner Meinung nach nur funktioniert, wenn es wirklich so ein Bestandteil für alle wird, für den Alltag, also halt für den Klinikalltag. (line 1294-1296)*
